# A truncated derivative of FGFR1 kinase cooperates with FLT3 and KIT to transform hematopoietic stem cells in syndromic and de novo AML

**DOI:** 10.1186/s12943-022-01628-3

**Published:** 2022-07-29

**Authors:** Baohuan Cai, Yun Liu, Yating Chong, Stephanie Fay Mori, Atsuko Matsunaga, Hualei Zhang, Xuexiu Fang, Chang-Sheng Chang, John K. Cowell, Tianxiang Hu

**Affiliations:** 1 Georgia Cancer Center, Augusta University, 1410 Laney Walker Blvd, Augusta, GA 30912 USA; 2 Department of Pediatrics, Tongji Hospital, Tongji Medical College, Huazhong University of Science and Technology, Wuhan, China; 3 Department of Geriatrics, Union Hospital, Tongji Medical College, Huazhong University of Science and Technology, Wuhan, China; 4 Department of Radiation Oncology, Tianjin Medical University Cancer Institute and Hospital, National Clinical Research Center for Cancer, Key Laboratory of Cancer Prevention and Therapy, Tianjin’s Clinical Research Center for Cancer, Tianjin, China

**Keywords:** FGFR1, FLT3, KIT, SCLL, Gene expression, Therapeutics, AML, GZMB

## Abstract

**Background:**

Myeloid and lymphoid malignancies associated with chimeric FGFR1 kinases are the hallmark of stem cell leukemia and lymphoma syndrome (SCLL). In all cases, FGFR1 kinase is constitutively phosphoactivated as a result of chromosome translocations, which lead to acquisition of dimerization motifs in the chimeric proteins. Recently, we demonstrated that these chimeric kinases could be cleaved by granzyme B to generate a truncated derivative, tnFGFR1, which localized exclusively into the nucleus and was not phosphorylated.

**Methods:**

Stem cell transduction and transplantation in syngeneic mice was used to assess the transforming ability of tnFGFR1 in bone marrow stem cells, and RPPA and RNA-Seq was used to examine the related signaling pathways and regulated target genes.

**Results:**

For the first time, we show that this non-classical truncated form of FGFR1 can independently lead to oncogenic transformation of hematopoietic stem cells in an animal model in vivo. These leukemia cells show a mixed immunophenotype with a B-cell B220 + Igm- profile in the majority of cells and Kit+ in virtually all cells, suggesting a stem cell disease. tnFGFR1, however, does not activate classic FGFR1 downstream signaling pathways but induces a distinct profile of altered gene expression with significant upregulation of transmembrane signaling receptors including FLT3 and KIT. We further show that de novo human AML also express tnFGFR1 which correlates with upregulation of FLT3 and KIT as in mouse leukemia cells. ChIP analysis demonstrates tnFGFR1 occupancy at the Flt3 and Kit promoters, suggesting a direct transcriptional regulation. Cells transformed with tnFGFR1 are insensitive to FGFR1 inhibitors but treatment of these cells with the Quizartinib (AC220) FLT3 inhibitor, suppresses in vitro growth and development of leukemia in vivo. Combined treatment with FGFR1 and FLT3 inhibitors provides increased survival compared to FGFR1 inhibition alone.

**Conclusions:**

This study demonstrates a novel model for transformation of hematopoietic stem cells by chimeric FGFR1 kinases with the combined effects of direct protein activation by the full-length kinases and transcriptional regulation by the truncated nuclear tnFGFR1 derivative, which is associated with GZMB expression levels. Genes significantly upregulated by tnFGFR1 include Flt3 and Kit which promote a leukemia stem cell phenotype. In human AML, tnFGFR1 activation leads to increased FLT3 and KIT expression, and higher FLT3 and GZMB expression levels are associated with an inferior prognosis. These observations provide insights into the relative therapeutic value of targeting FGFR1 and FLT3 in treating AML with this characteristic gene expression profile.

**Supplementary Information:**

The online version contains supplementary material available at 10.1186/s12943-022-01628-3.

## Background

The fibroblast growth factor receptor-1 (FGFR1) kinase has a profound role during embryonic development and its expression is tightly coordinated, particularly in the development of the hematopoietic system where it influences maintenance of the stem cell compartment ensuring normal early hematopoiesis [1]. This critical role during development in the regulation of proliferation and differentiation, however, has been subverted in a variety of cancer cell types to promote uncontrolled proliferation [2]. In the stem cell leukemia/lymphoma syndrome (SCLL), structural chromosome changes juxtapose dimerization motifs adjacent to the FGFR1 kinase domain leading to ligand independent, constitutive activation of FGFR1 kinase [3, 4]. As a result, FGFR1-dependent activation of a variety of cytoplasmic proteins such as PLCG2, SRC [5], SHP2 [6] and GRB2 [7] occurs, which in turn promote proliferation through, for example, ERK/MAPK and AKT signaling cascades. FGFR1 also leads to significant reorganization of transcription factor expression affecting metabolic, cell mobility and adhesion characteristics [8]. Targeting FGFR1 activation with a variety of drugs has proved effective in suppressing leukemia cell growth in vitro and leukemogenesis in mouse models of SCLL [9, 10], as well as in SCLL patients [11, 12] and hence is potentially an effective way of treating this disease using targeted therapies. Resistance to these inhibitors, however, can develop [13, 14], in part due to mutation in the kinase domain, which prevents binding of drugs targeting its phosphoactivation [15].

FGFR1, upon ligand activation, normally recruits intermediate signaling molecules [1] which activate cytoplasmic proteins to promote proliferation, suppress apoptosis and maintain the stem cell phenotype in SCLL. Activation involves phosphorylation of critical tyrosine residues within the kinase domain and their mutation suppresses activation and subsequent signaling [13]. It has been shown, however, that the full-length FGFR1 chimeric proteins can be truncated at a site immediately proximal to the kinase domain [16, 17]. This truncated form (tnFGFR1), unlike the fusion kinases, is not phosphorylated so cannot directly activate other proteins in the cell and, unlike most of the chimeric fusion kinases, is located exclusively in the nucleus. The lack of a dimerization motif in tnFGFR1 implies that it exerts its effect as a transcription factor or co-factor and, in focused experiments, we showed that it can occupy sequences in the c-Myc promoter [17]. There has, however, been no direct evidence implicating tnFGFR1 in leukemia or cancer initiation and progression.

We now show that tnFGFR1 can transform primary bone marrow stem cells to produce a stem cell leukemia with a mixed B-lymphoma/stem cell immunophenotype using transduction and transplantation of primary bone marrow in mouse models. These cells show upregulation of Flt3 and Kit, which was also shown in an analysis of primary human AML cells. ChIP analysis indicates a direct role for tnFGFR1 in the transcriptional regulation of Flt3 and Kit. Cells expressing tnFGFR1 are insensitive to FGFR1 kinase inhibitors, but because of the upregulation of Flt3 in these cells they are sensitive to AC220, providing a potential treatment for this lethal disease.

## Materials and methods

### In vitro BaF3 cell and bone marrow transformation assays

Transduction of BaF3 and bone marrow cells were performed using retroviruses containing various construct variants using the empty pMIG vector as the control. Details of this vector system can be found in the study by Ren et al. [18]. The transformation ability of these variants was measured by their ability to generate IL3 independence of transduced BaF3 cells. Analysis of transformation of sorted GFP+ mouse bone marrow (BM) cells was performed using plating assays as described previously [19]. Briefly, transduced bone marrow cells were isolated using cell sorting based on expression of GFP from the targeting vectors. Cells were then diluted to different concentrations in 24-well plates and cultured in RPMI media supplemented with 10% fetal bovine serum. After 3 weeks, cells were harvested from each well and scored as positive if the cell numbers exceeded 1 × 10^6^/ml.

### Bone marrow transduction and transplantation in vivo

All animal experiments were performed under an approved protocol (IACUC 2008–0155) from the Augusta University Institutional Animal Care and Use Committee. 6–8-week-old female BALB/c mice were used for all engraftment experiments. Donor BM cells from BALB/c mice were isolated and transduced with various constructs prepared in the MIG retroviral vector as described previously [18–20], then 5 × 10^6^ cells were transplanted into lethally irradiated (800 cGy), syngeneic hosts via tail vein injection. Tumor cell engraftment and progression was monitored weekly beginning two weeks after i.v. injection by examination of the levels of the GFP+ population in peripheral blood using standard flow cytometry analysis. Antibodies used were, B220-APC (#553092), IgM-PE (#553409, CD4-APC (#553051), CD8-PE/Cy7 (552877), Gr1-PE (553128), Mac1-PerCP/Cy5.5 (#550993), Sca1-PE/Cy7 (558162), Kit-APC (553356) and Flt3-APC (#560718) from BD Biosciences (Franklin Lakes, NJ). For secondary and subsequent sequential transplantations, 1–2.0 × 10^6^ BM cells from primary and subsequent diseased mice, respectively, were again transferred by tail vein injection into sub-lethally irradiated (600 cGy) female BALB/c recipient mice.

### Analysis of human AML

Human AML samples were obtained from the Georgia Cancer Center Biorepository which were collected under approved IRB protocol 611,132–42 and supplied as anonymized samples through an honest broker agreement. The PDX samples used were obtained from archival samples generated as described previously [10]. The GSE37642 cohort of primary human AML samples [21] was analyzed using the R2 Genomics Analysis and Visualization Platform (http://r2.amc.nl). The AML patient samples in the Cancer Genome Atlas (TCGA) PanCancer Atlas were analyzed and visualized using the cBioPortal platform [22, 23].

### Inhibitor treatment

Drugs used in these studies were: GZMB Inhibitor II (CAS 1258003–96-1, Calbiochem), Infigratinib (BGJ398) and Quizartinib (AC220) (Selleckchem, Houston, TX). For in vitro drug inhibition assays, cells were treated at the indicated concentrations of BGJ398 or AC220 for 72 h, followed by cell viability assays using the CellTiter-Glo Luminescent Cell Viability Assay (Promega, Madison, WI). Cell cycle progression and apoptosis levels were measured as previously described [6] using Hoechst 33342 (Invitrogen, Waltham, MA) and Annexin V (Biolegend, San Diego, CA) staining respectively, according to the manufacturer’s protocol and analyzed using NovoCyte Quanteon flow cytometry (Agilent, Santa Clara, CA). All drug treatment experiments were repeated at least three times and representative results are presented.

For in vivo FLT3 inhibitor treatments, 2 × 10^6^ BM cells (of which 90% are GFP+ leukemia cells) from the tnFGFR1 mice with the disease were transplanted into recipients through tail vein injection. Expansion growth was allowed for one week before treatment with either inhibitor or vehicle. The randomly grouped mice were then treated daily from days 7 to 15 with either vehicle or the AC220 inhibitor at 5 or 10 mg/kg body weight via oral gavage. For combinational treatment, the same number of GFP+ leukemia cells from the BCR-FGFR1 mice were transplanted *i.v*. The recipient mice were then treated with either vehicle, BGJ398 at 30 mg/kg, AC220 at 10 mg/kg, or BGJ398 and AC220 in combination. Treatment was discontinued after all the mice in the vehicle group had died of leukemia. Flow cytometry was performed to evaluate the engraftment ratio of GFP+ leukemia cells in the peripheral blood and mice were sacrificed in the presence of advanced disease.

### Molecular analyses

For Western blotting, cells were lysed in ice-cold T-PER™ Tissue Extraction Reagent (Thermo Scientific), 0.5 M EDTA, and a mixture of proteases inhibitors. 20 μg protein samples were measured using the Pierce™ BCA protein Assay kit and analyzed by SDS-PAGE followed by transfer to nitrocellulose (Bio-Rad) membrane using the Bio-Rad gel and transfer apparatus. Membranes were incubated in 5% whole milk for 1 hour at room temperature, washed with TBS-T, followed by incubation with the primary antibody (as specified) overnight at 4 °C. The membrane was washed with TBS-T and then incubated with the appropriate secondary antibodies at room temperature for 1 hour and the immunocomplexes were visualized using the ECL Western Blotting substrate or SuperSignal™ West pico PLUS chemiluminescent Substrate or SuperSignal™ West Femto Maximum Sensitivity Substrate from Thermo Sicentific, based on their expression levels. The antibodies used for western blotting were; FGFR1 (Cell signaling, #9740), p-FGFR1 (Abcam, ab59194), β-Actin (Cell signaling, #5125), Gzmb (ABclonal, A2557), p-Stat3 (Cell Signaling, #9134), Stat3 (Cell Signaling, #4904), p-S6 (Cell Signaling, #2211), S6 (Cell Signaling, #2217), p-Shp2 (Cell Signaling, #3751), Shp2 (Cell Signaling, #3397), Kit (Cell Signaling, #3074), p-Flt3 (Cell Signaling, #4577), Flt3 (Cell Signaling, #3462).

Bone marrow cells, which contain > 90% GFP+ primary leukemia cells, were used directly for RNA-seq based gene expression profiling as described previously [8]. ChIP-qPCR was performed as described previously [17, 24] using an antibody (ab58516, Abcam) specific for truncated FGFR1 and the Myc-Tag antibody (#2276S, Cell Signaling). shRNA knockdown experiments were performed as described previously [17]. qRT-PCR, plasmid preparation and transfection, and cell culture followed standard procedures that have been described in detail previously [20].

### Reverse-phase protein array (RPPA) analysis

In this assay, expression levels of both proteins and protein modifications such as phosphorylation broadly implicated in cancer development were determined in cell lysates from the MC and KO BBC2 cells. Briefly, denatured cellular lysates were arrayed as microdots on nitrocellulose coated glass slides at various concentrations and probed individually with ~ 304 highly specific antibodies that recognize mouse proteins involved in most common signaling pathways implicated in cancer development and progression and which have been validated for RPPA. Various control lysates and peptides are also present on the array. The RPPA assay was performed by the Functional Proteomics RPPA Core Facility at MD Anderson Cancer Center - https://www.mdanderson.org/research/research-resources/core-facilities/functional-proteomics-rppa-core.html, − where the complete list of antibodies used is available. Signals were captured by tyramide dye deposition and a DAB colorimetric reaction, quantified and normalized as described previously [25].

### Statistical analyses

All statistical analysis for pairwise comparisons were performed using the Student’s T test. For the combinational drug treatment studies, mouse survival, spleen weight and white blood cell count data was analysed using ANOVA with Bonferroni corrections. *p ≤ 0.05, **p ≤ 0.01, ***p ≤ 0.001, ****p = 0.0001. ns = not significant. Error bars represent standard deviation.

## Results

### tnFGFR1 does not acutely transform BaF3 or primary bone marrow cells

**Fig. 1:**
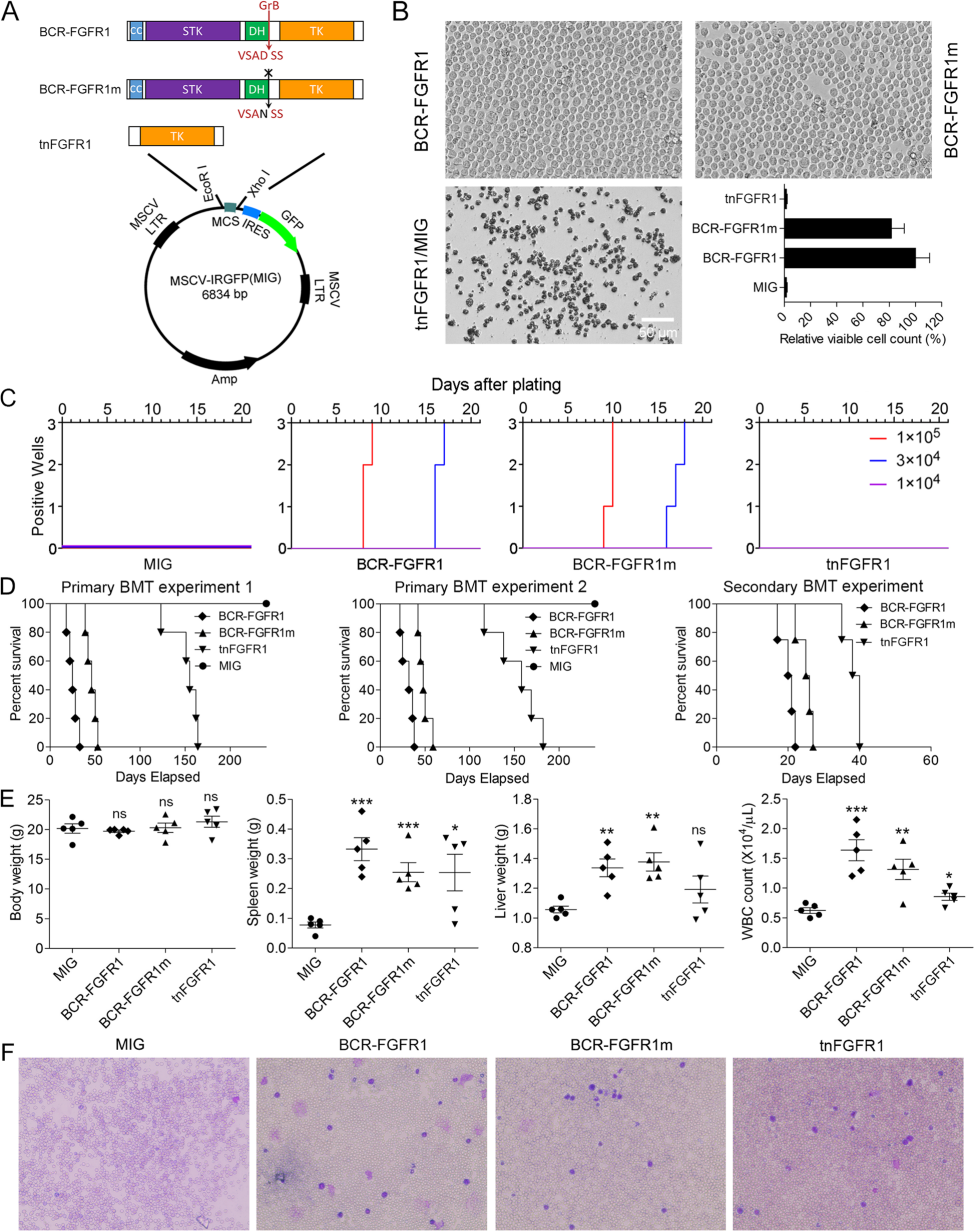
Assessment of the oncogenic transformation ability of tnFGFR1. Schematic of the retroviral constructs used in transformation assays indicating the location of the mutation in the granzyme-B recognition sequence and truncation of the BCR chimeric kinase (**A**). Acute transformation potential of these three constructs is shown in (**B**) where the BCR-FGFR1 kinase and its mutant derivative (*N* = 3) can competently transform BaF3 cells to IL3 independence compared with the tnFGFR1 construct and the empty vector, which cannot. In a plating assay using normal murine BM cells transduced with the different constructs (*N *= 3), tnFGFR1 failed to promote proliferation of BM cells, compared with the BCR-FGFR1 and BCR-FGFR1m constructs (**C**). Kaplan-Meier survival curves for cohorts of mice (*N* = 5) receiving transplants of stem cells transduced with either the BCR-FGFR1 chimeric gene or its mutant form, compared with stem cells transduced with either the empty vector or the tnFGFR1 construct (**D**). In two independent experiments, while both the BCR-FGFR1 construct and its mutant derivative results in rapid onset (30–50 days) of leukemia, the cells transduced with the empty vector do not develop disease. Cells transduced with tnFGFR1 develop disease but with a significantly extended latency period (150–160 days). When the cells from the primary experiment were transplanted into secondary hosts, while the latency period was significantly reduced, the relative latency period between the full-length kinases and tnFGFR1 is maintained (**D**). Scatter plots for individual mice (*N* = 5) in the four subgroups (**E**) demonstrates that leukemia development is associated with increased liver and spleen weight as well as WBC count compared with the MIG control. The two spleens that showed low overall weight were derived from the mice that died early in the experiment probably because of high involvement of the BM before significant mobilization of leukemic stem cells to the peripheral circulation. Analysis of blood smears from xenografted mice transfected with each of the constructs supports the leukemia diagnosis (**F**). ns = not significant. *p ≤ 0.05, **p ≤ 0.01, ***p ≤ 0.001

BaF3 is an IL3 dependent, murine, pro-B cell line that has been used extensively in the study of leukemogenesis and is a well-known assay for evaluating the ability of genes to transform BaF3 cells into IL3 independence as an indication of oncogenic potential [26]. Using the tnFGFR1 construct created in the MIG retroviral vector (Fig. 1A), we noted that BaF3 cells cannot be acutely transformed to IL3 independence in this short-term survival assay. In contrast, the parental BCR-FGFR1 chimeric kinase and a mutant form of the gene in which the granzyme-B (GZMB) recognition site was mutated (BCR-FGFR1m) such that tnFGFR1 cannot be generated (Fig. 1A) successfully transformed BaF3 cells to IL3 independence, demonstrating that BCR-FGFR1m is as effective an oncogene as the parental gene in this assay (Fig. 1B). To investigate the effect of tnFGFR1 on primary cells, BM cells were transduced with retroviruses expressing either BCR-FGFR1/GFP, BCR-FGFR1m /GFP, tnFGFR1/GFP or GFP alone. GFP-positive cells were then flow sorted and cultured in vitro. Cells transduced with both BCR-FGFR1 and BCR-FGFR1m showed rapid cell proliferation at the two higher initial concentrations, while cells transduced with the tnFGFR1 construct did not show any evidence of growth or survival (Fig. 1C). Thus, from these transformation assays it does not appear that tnFGFR1 is acutely oncogenic in this short-term assay.

### tnFGFR1 can transform mouse hematopoietic stem cells independently in vivo

The prevailing hypothesis for chimeric kinase transformation of stem cells is that, through its dimerization, the FGFR1 kinase domain becomes phosphoactivated which, in turn, leads to activation of downstream oncogenic signaling pathways [5–7, 19, 20, 27]. Since tnFGFR1 does not have the dimerization motif present in the chimeric kinases, and is not phosphorylated [17], the question arose whether it could act independently as an oncogene to transform primary hematopoietic stem cells in vivo in the longer term. To investigate this potential, therefore, we used the in vivo bone marrow transduction and transplantation assay described previously [19]. Using retroviral constructs, murine hematopoietic stem cells were transduced ex vivo and transplanted into lethally irradiated syngeneic BALB/C hosts. Both the BCR-FGFR1 and BCR-FGFR1m transduced cells led to a typically [28] rapid (< 21 days) onset of leukemogenesis (Fig. 1D). In contrast to the BaF3 assay results, however, tnFGFR1 transduced cells also developed leukemia, but only after a relatively long (~ 150 days) latency period (Fig. 1D). These experiments were performed on two occasions with the same result. When the tnFGFR1 transformed cells were transplanted into secondary hosts, the latency period for disease development, although shorter (~ 40 days) than that seen in the primary transplant, was again relatively long compared with the full length and mutant chimeric kinases (Fig. 1D). The in vivo leukemogenesis analysis was supported by the increased spleen and liver weights as well as the white blood cell count at the time of sacrifice in the primary transplanted mice after transduction with the various constructs (Fig. 1E). Compared to the wild-type BCR-FGFR1 and BCR-FGFR1m, the WBC levels in tnFGFR1 leukemic mice show a moderate increase, suggesting less mobilization of leukemia cells from the bone marrow into the peripheral circulation. The increased WBC count was also supported by Wright-Giemsa staining of blood smears from these mice (Fig. 1F). In addition, H&E staining of spleen tissue sections also confirmed high levels of transformed leukemia cells in these mice (Supplement Figure 1).

### tnFGFR1 transformed stem cells have a biphenotypic immunophenotype

**Fig. 2:**
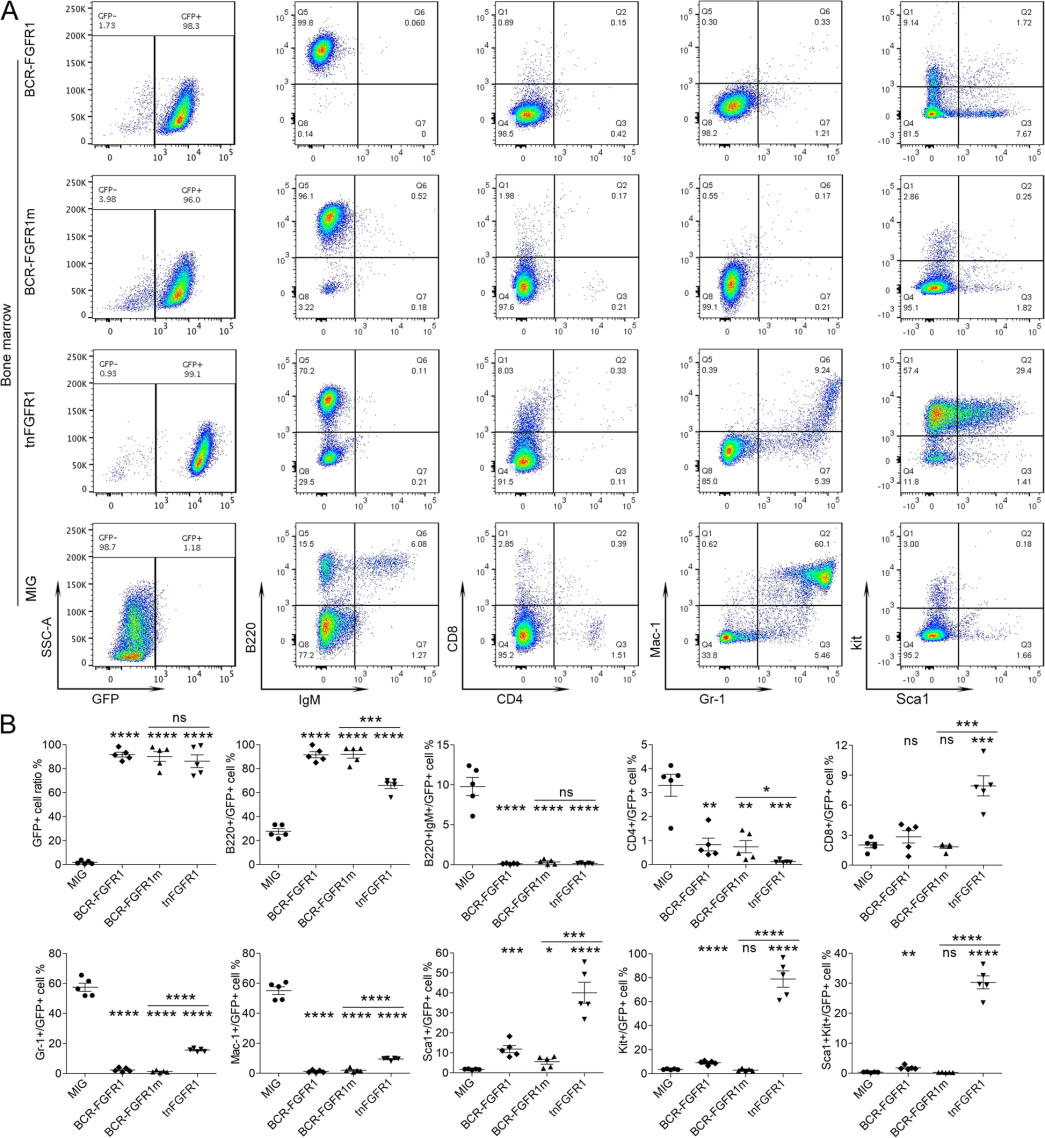
tnFGFR1 promotes leukemia development with a stem cell immunophenotype. Representative flow cytometric profiles of the bone marrow cells from the mice transplanted with various derivative constructs of the BCR-FGFR1 kinase (**A**) at the time of sacrifice show extensive GFP-positive cells in all cases, demonstrating leukemic development compared with the MIG control which are GFP-negative (normal) cells. Analysis of cell lineage-specific markers demonstrates low levels (< 10%) of T-cell (CD8/CD4) and myeloid (Mac1/Gr1) markers but high-level presence of the B220 B-cell marker in cells transduced with the full length chimeric kinases as well as the tnFGFR1 transduced cells. Analysis of stem cell markers KIT and SCA1, however, indicate that while the BCR-FGFR1 kinases and its mutant derivative are mostly negative, the cells from the tnFGFR1 transduced mice predominantly express KIT with a small subgroup also expressing SCA1. Quantitative scatter plots for each marker in each individual mouse within the subgroups (*N* = 5) compared with the MIG transfected control cells are shown in (**B**). ns = not significant. *p ≤ 0.05, **p ≤ 0.01, ***p ≤ 0.001, ****p ≤ 0.0001.. These values represent pairwise comparison between individual oncogenic chimeric kinases and the normal MIG group in each case

Transduced bone marrow cells co-express GFP from the MIG transduction vector [19], allowing specific isolation and analysis of transduced cells. Since the MIG transfected cells do not transform, the samples from these recipient mice are GFP-negative and so represent those seen in normal mice. Flow cytometric analysis showed that the GFP+ leukemic cells derived from the bone marrow of the tnFGFR1 transduced mice were mostly negative for the T-cell and myeloid lineage markers analyzed (Mac1, Gr1, CD4, CD8,) but showed the same high-level B220+ B-cell phenotype seen in the BCR-FGFR1 transformed cells (Fig. 2). Importantly, the tnFGFR1 transformed cells also showed a predominant KIT+ immunophenotype with a small Sca1+ subpopulation, suggesting a relatively pure population of cells with a stem cell immunophenotype (Fig. 2). In contrast, cells transformed with either the parental full-length kinase or the BCR-FGFR1m kinase predominantly showed the previously reported B220 + IgM- immunophenotype [28] with small subpopulations that express Kit and/or Sca1. Analysis of the spleen cell from the same strains showed identical immunophenotypes (Supplement Figure 2). It appears, therefore, that tnFGFR1 can promote expansion of leukemia stem cells with a B-cell/stem cell immunophenotype, which is not typically seen for the various chimeric kinases.

**Fig. 3:**
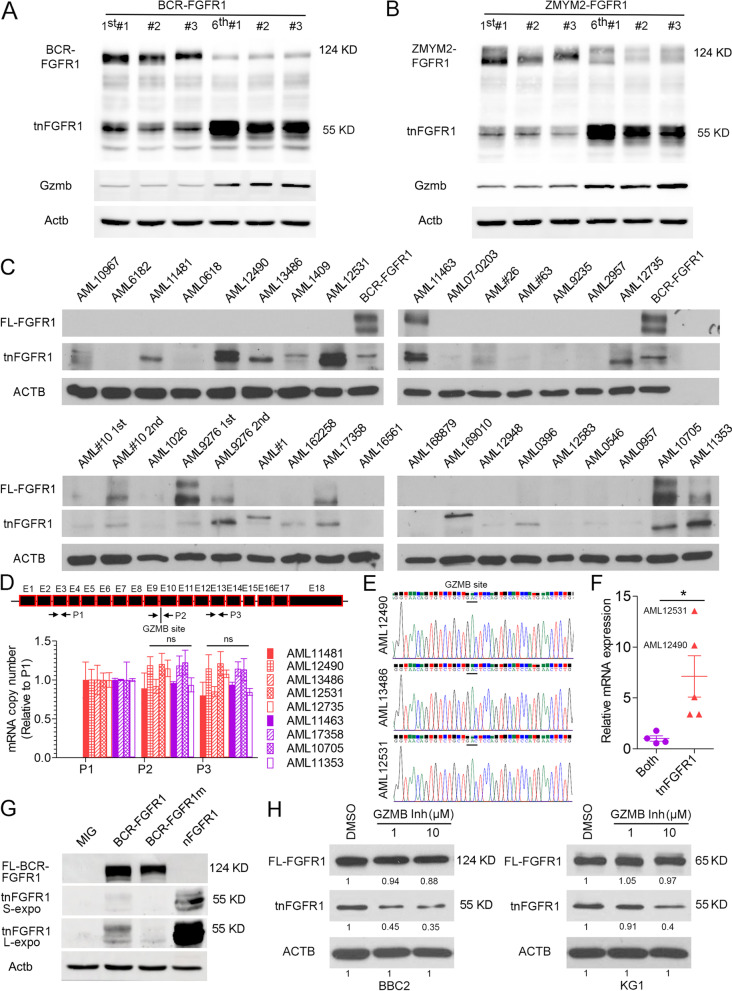
tnFGFR1 is expressed widely in primary leukemia cells from mouse models and AML patients and is produced by GZMB cleavage of the full-length FGFR1. Western blot analysis of mice (*N* = 3) with primary transduced cells shows the full length kinase is expressed at high levels relative to the truncated tnFGFR1 in both BCR-FGFR1 (**A**) and ZMYM2-FGFR1 (**B**) murine leukemic cells. After serial transplantation of these cells from the primary mice through 6 cycles, the serially transplanted leukemia cells consistently demonstrate dominant expression of the tnFGFR1 derivative. In these cohorts there is increased expression of GzmB that is proportional to the increase in tnFGFR1 levels. Analysis of 29 primary AML bone marrow samples and 4 patient-derived xenograft (PDX) samples from immune compromised mice (**C**) demonstrates the presence of the full length FGFR1 kinase in specific cases, most of which also show the presence of the truncated kinase. In several other cases, there is predominant expression of the truncated kinase. For AML#10 and AML9276, the second transplant shows increased levels of FGFR1 proteins as seen in the syngeneic transplants, with AML9276 showing preferable enrichment of tnFGFR1. BaF3 cells transformed with the full length kinase (BCR-FGFR1) are used as molecular size controls. qRT-PCR analysis using primers targeting sequences in the proximal region of FGFR1 (P1), across the granzyme B cleavage site (P2) and within the tnFGFR1 derivative (P3) in a subset of nine of the most representative human AML samples (**D**) shows no significant difference in the relative copy number of different regions of FGFR1 mRNA between the tnFGFR1 exclusive samples and samples expressing both the full-length and truncated FGFR1 proteins. Sanger sequencing across the granzyme B cleavage site (**E**) from three AML that show high levels of tnFGFR1 protein show no alternative splicing around the GZMB site. qRT-PCR analysis of GZMB mRNA levels in the same samples (**F**) shows that higher levels of tnFGFR1 protein is correlated with high levels of GZMB expression. Mutation of the GZMB recognition site prevents tnFGFR1 production in BaF3 cells transformed with BCR-FGFR1m, compared with its parental BCR-FGFR1 transformed cells (**G**). Longer exposure times (L-expo) reveals tnFGFR1 in the cells transduced with BCR-FGFR1 but not BCR-FGFR1m. When mouse BBC2 and human KG1 SCLL cell lines are treated with GZMB Inhibitor II, quantitation of the protein levels normalized to actin levels (ACTB) shows the production of tnFGFR1 is decreased accordingly with increased inhibitor concentration (**H**). ns = not significant. *p ≤ 0.05

The extended latency of leukemia progression and stem cell like immunophenotype in the tnFGFR1 primary leukemia model suggested a potential direct role of tnFGFR1 in promotion and maintenance of the leukemia stem cell population. When the expression of tnFGFR1 was examined in bone marrow cells following serial transplantation of leukemias driven by BCR-FGFR1 (Fig. 3A) and ZMYM2-FGFR1 (Fig. 3B), the primary leukemia cells showed a predominant expression of the full-length kinase, with relatively low levels of tnFGFR1. In contrast, the leukemia cells from the 6th generation of transplantation expressed much higher tnFGFR1 levels, with a relatively reduced expression of the full-length FGFR1 fusion kinases, indicating an enrichment of the tnFGFR1 expressing leukemia stem cells during the progressive transplantations.

### tnFGFR1 is frequently generated in human AML cells

While FGFR1 chimeric kinases are specifically associated with SCLL, our previous studies in primary human AML demonstrated increased expression of FGFR1 in up to 20% of de novo human AML samples [10]. To evaluate the presence of tnFGFR1 in human AML, we surveyed its expression levels in 38 archival primary AML bone marrow samples and 4 patient-derived xenograft (PDX) samples (Fig. 3C, Supplement Figure 3A) that were previously established in immunocompromised mice [10]. As suggested from our previous observations [10], 9.52% of these primary human samples (4/42; AML11463, AML17358, AML10705 and AML11353) as well as cells from four PDX models AML#10 1st, AML#10 2nd, AML9276 1st and AML9276 2nd expressed a full length FGFR1 kinase. Consistent with the observation in the mouse models, all of these samples also expressed the truncated tnFGFR1. Surprisingly, an additional 15 samples (35.71%; AML10967, AML11481, AML12490, AML13486, AML1409, AML12531, AML#26, AML12735, AML#1, AML162258, AML169010, AML0396, AML0546, AML1049, AML#40) apparently only expressed tnFGFR1, albeit at variable levels, without the presence of detectable levels of the full length FGFR1 kinase (Fig. 3C, Supplement Figure 3A), Of note is that in the 4 AML PDX samples (AML#10 and AML9276), the bone marrow samples from the 2nd serial engraftment of AML9276 showed preferential increases in tnFGFR1 expression, similar to the observation in the serial transplantation in the mouse models, implying a potential role of tnFGFR1 in maintaining the AML stem cell phenotype.

The observation from western blotting (WB) analysis (Fig. 3C, Supplement Figure 3A) showed that in some cases the truncated FGFR1 protein was present apparently in the absence of the full-length protein, which raised the issue of whether this is due to the rapid turnover of the full length protein by GZMB cleavage or as a result of alternative splicing at the mRNA level. To investigate this issue, we performed qRT-PCR analysis of the RNA isolated from AML patient samples used for the WB in Fig. 3C for which high quality RNA was available. In this study we designed primers around exon 3 of FGFR1 (P1), across the granzyme B cleavage site (P2) and around exon 13 in a region retained in the tnFGFR1 variant. In the qPCR analysis, a 1:1:1 amplification ratio of all three primers would indicate the presence of a full length FGFR1 mRNA transcript. Alternatively, a 1:1:2 ratio would indicate the presence of two transcripts, one from a full length mRNA and one from a spliced tnFGFR1 transcript. As shown in Fig. 3D, the relative expression levels of P2 and P3 in the 9 samples analyzed were normalized to their respective levels of the P1 amplicon. When the levels of P2 and P3 amplicons were compared between samples which clearly expressed both full length and truncated proteins and those that apparently showed only the truncated protein, there was no significant difference in mRNA copy number for each of the internal sites of FGFR1, demonstrating that there is no independent short tnFGFR1 transcript. Furthermore, Sanger sequencing of the P2 mRNA products spanning the GZMB cleavage site from three samples where the full-length protein was not detected by WB, showed no alternative splicing around this region (Fig. 3E). Together, these results indicate that only the full-length FGFR1 mRNA is present in these samples (Fig. 3D and E).

### tnFGFR1 is generated by GZMB cleavage of the full -length fusion kinase

We previously demonstrated that tnFGFR1 in SCLL is generated through GZMB cleavage [17]. When GZMB expression levels were analyzed in samples from serial transplantation in vivo, the significantly higher levels of tnFGFR1 expression in the leukemia cells from the 6th transplantation were reflected in an increased GZMB expression level in the same cells (Fig. 3A and B), compared with the primary transplantation. These observations are consistent with the idea that tnFGFR1 levels increase proportionately with GZMB enzyme activity. Analysis of GZMB transcript levels using qRT-PCR (Fig. 3F) in this cohort showed significantly increased mRNA expression levels in all cases where only the tnFGFR1 protein was identified by WB compared with those where both protein isoforms were detected and further, the highest expression levels were shown in the two samples that showed highest levels of tnFGFR1 protein (AML12490 and AML12531). Overall, this analysis demonstrates that the truncated FGFR1 protein is not a result of alternative splicing at the mRNA level but at the level of protein cleavage and that the higher the GZMB levels the more prevalent the tnFGFR1 protein. Consistently, the serial PDX samples show unbiased enrichment of the full-length FGFR1 transcript (Supplement Figure 3B). AML9276, which displays a preferential increases in tnFGFR1 protein expression, with reduced full-length FGFR1 after serial transplantation, shows significantly higher upregulation of GZMB transcription (Supplement Figure 3B).

Using the BaF3 cell lines transduced with different variant constructs, we further confirmed that mutation in the GZMB recognition site prevented the production of tnFGFR1 (Fig. 3G). An exclusive expression of tnFGFR1 was observed in the cells transduced with this truncated derivative. With increased exposure, a clear signal from tnFGFR1 can be seen in the cells expressing BCR-FGFR1, but not in the BCR-FGFR1m derivative (Fig. 3G). To complement these observations, we also used GZMB inhibitor II (previously called Ac-IETD-CHO [29]) to pharmacologically inhibit its activity in SCLL cell lines BBC2 and KG1 (Fig. 3H), where there was a dose-dependent inhibition of the production of tnFGFR1, further confirming the role of GZMB in the generation of the truncated nuclear tnFGFR1 derivative in both human and mouse models.

### tnFGFR1 does not appear to activate classic FGFR1 signaling intermediates

**Fig. 4:**
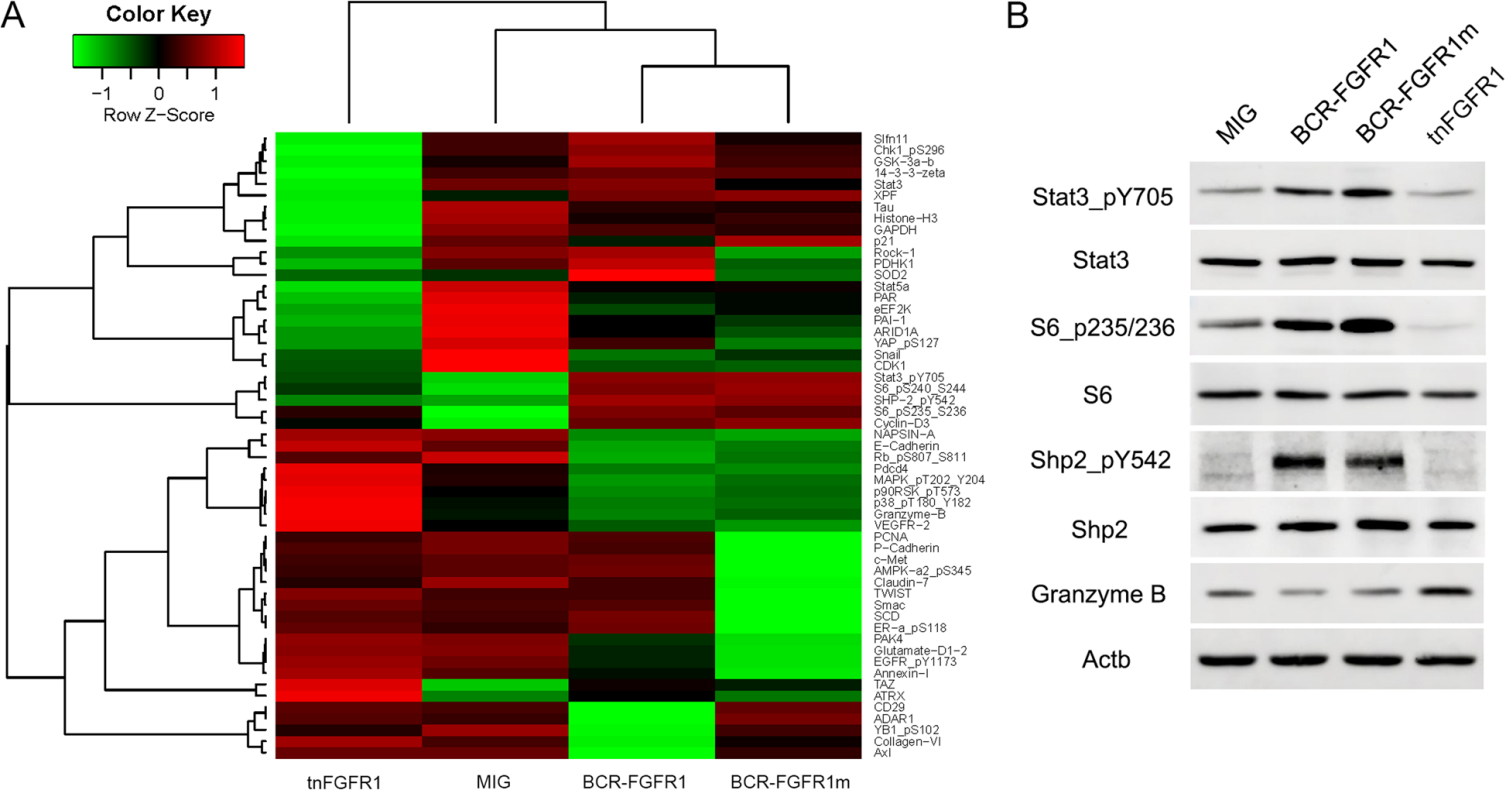
The truncated tnFGFR1 shows a distinct profile of oncogenic signaling compared with the full length chimeric kinases. RPPA analysis of BaF3 cells transduced with either empty vector (MIG) or tnFGFR1 and maintained on IL3, or transformed with the BCR-FGFR1 chimeric kinase or its GZMB site mutated version (**A**). Selected preferentially activated pathways identified by RPPA are further validated by western blotting (**B**)

To investigate changes in signaling pathways resulting from tnFGFR1 expression, we compared Reverse Phase Protein Array (RPPA) analysis [30] of BaF3 cells transduced with either the full length or mutant BCR-FGFR1 kinases with those transduced with either tnFGFR1 or the empty vector. This assay measures protein levels for a series of 304 protein targets implicated in cancer development. As shown in Fig. 4A, the parental BCR-FGFR1 and the BCR-FGFR1m variant displayed a similar expression profile reflecting classical FGFR1 signaling pathways, such as activated Stat3_pY705, SHP_pY542, Cyclin-D3, S6_pS235/236 and S6_pS240/244. In contrast, tnFGFR1-expressing cells did not show activation of any of these signaling intermediates. Instead, tnFGFR1 preferentially activated Pdcd4, MAPK_pT202_Y204, p90RSK_pT573, p38_PT180_Y182, GZMB and VEGFR-2. The differential activation of some of these pathways was further confirmed by western blot analysis (Fig. 4B). Of particular interest was the upregulation of GZMB in tnFGFR1 expressing cells compared with the other transduced cells, suggesting a possible positive feedback regulation between tnFGFR1 and GZMB (Fig. 4A and B), to promote the production of the tnFGFR1 derivative in these cells. The low level of GMZB in the full-length BCR-FGFR1 kinases correlates with high levels of the full length chimeric FGFR1 kinase and relative low expression of tnFGFR1 in these cells.

### Identification of genes regulated by tnFGFR1 during leukemogenesis

**Fig. 5:**
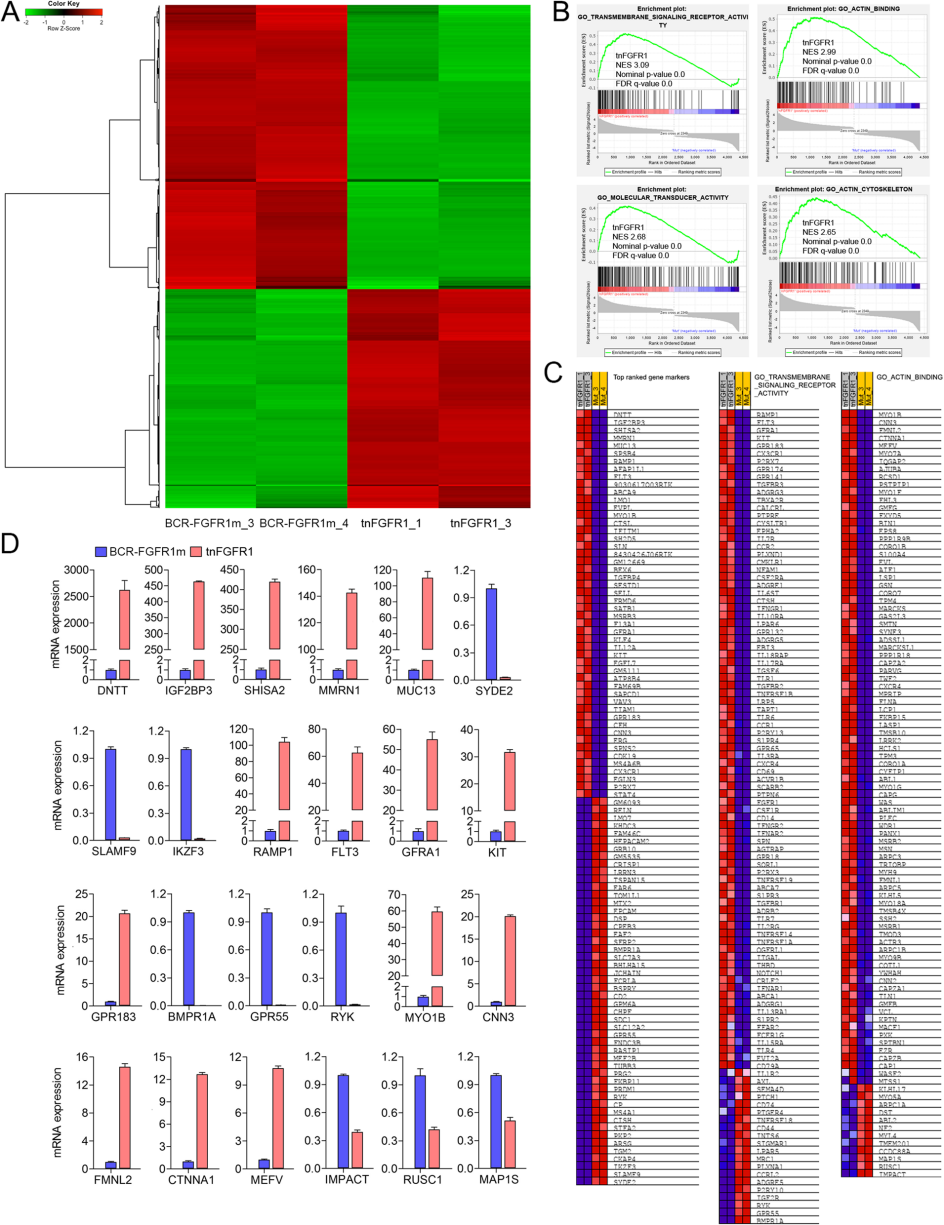
tnFGFR1 regulates a specific subset of genes during leukemogenesis. RNA-Seq analysis of leukemic cells from the bone marrow from mice transplanted with stem cells transduced with either tnFGFR1 or the mutant BCR-FGFR1m (*n* = 2) construct identifies specific patterns of expressed genes as shown in the heat map in (**A**). GSEA analysis (**B**) identifies significant enrichment for differential changes in pathways involved in receptor signaling, actin and cytoskeleton, and molecular transduction. The comparison in expression changes of genes in related pathways using qPCR is shown in (**C**) and qPCR verification (*N* = 3) of several of the most highly differentially expressed genes is shown in (**D**)

To obtain a more detailed understanding of genes that might be specifically regulated by tnFGFR1 during leukemogenesis, we performed RNA-Seq analysis in a comparison between tnFGFR1 transformed primary leukemia cells and those transformed with the BCR-FGFR1m construct in which the truncated FGFR1 derivative is not generated. Genes activated in the tnFGFR1 transformed cells should be due to direct activation by the truncated kinase but the cytoplasmic activation events would not occur. Bone marrow cells from mice in which leukemogenesis was induced in hematopoietic stem cells were isolated from two different animals and RNA-Seq performed as described previously [8]. Data was processed using conventional approaches based on FPKM scores and the comparison generated a heat map of differentially expressed genes (Fig. 5A). The most significant GO functional groups identified using GSEA analysis included transmembrane signaling receptor activity, molecular transducer activity, actin binding and actin cytoskeleton (Fig. 5B). The rank order of genes upregulated as a result of tnFGFR1 expression is shown in Fig. 5C and their upregulation was confirmed by qPCR for the five (DNTT, IGF2BP3, SHISA2, MMRN1 and MUC13) most highly overexpressed genes (Fig. 5D). The three most under expressed genes in tnFGFR1 transformed cells (SYDE2, SLAMF9 and IKZF3) were also verified by qPCR (Fig. 5D). Similar qPCR verification was performed for genes in the GO-transmembrane signaling receptor activity and actin binding subgroup (Fig. 5D). Of note, the levels of expression changes in the genes showing under expression in all groups was significantly lower than those seen in the overexpressed genes, demonstrating a dominant role for tnFGFR1 in gene activation. When these gene expression profiles were compared with normal lineage negative Kit-expressing murine stem cells (Supplement Figure 4), there was a subgroup of genes exclusively expressed in the tnFGFR1 transformed cells and a different subgroup specifically expressed in the BCR-FGFR1m transformed cells. Analysis of the top ranked GO functional groups in these subpopulations of genes indicated that BCR-FGFR1m appears to specifically regulate cell proliferation and survival pathways, while the tnFGFR1 regulated subgroup of genes were specifically involved in maintaining an undifferentiated stem cell phenotype (Supplement Table 1).

### FLT3 and KIT are direct target genes regulated by tnFGFR1 during leukemogenesis

**Fig. 6:**
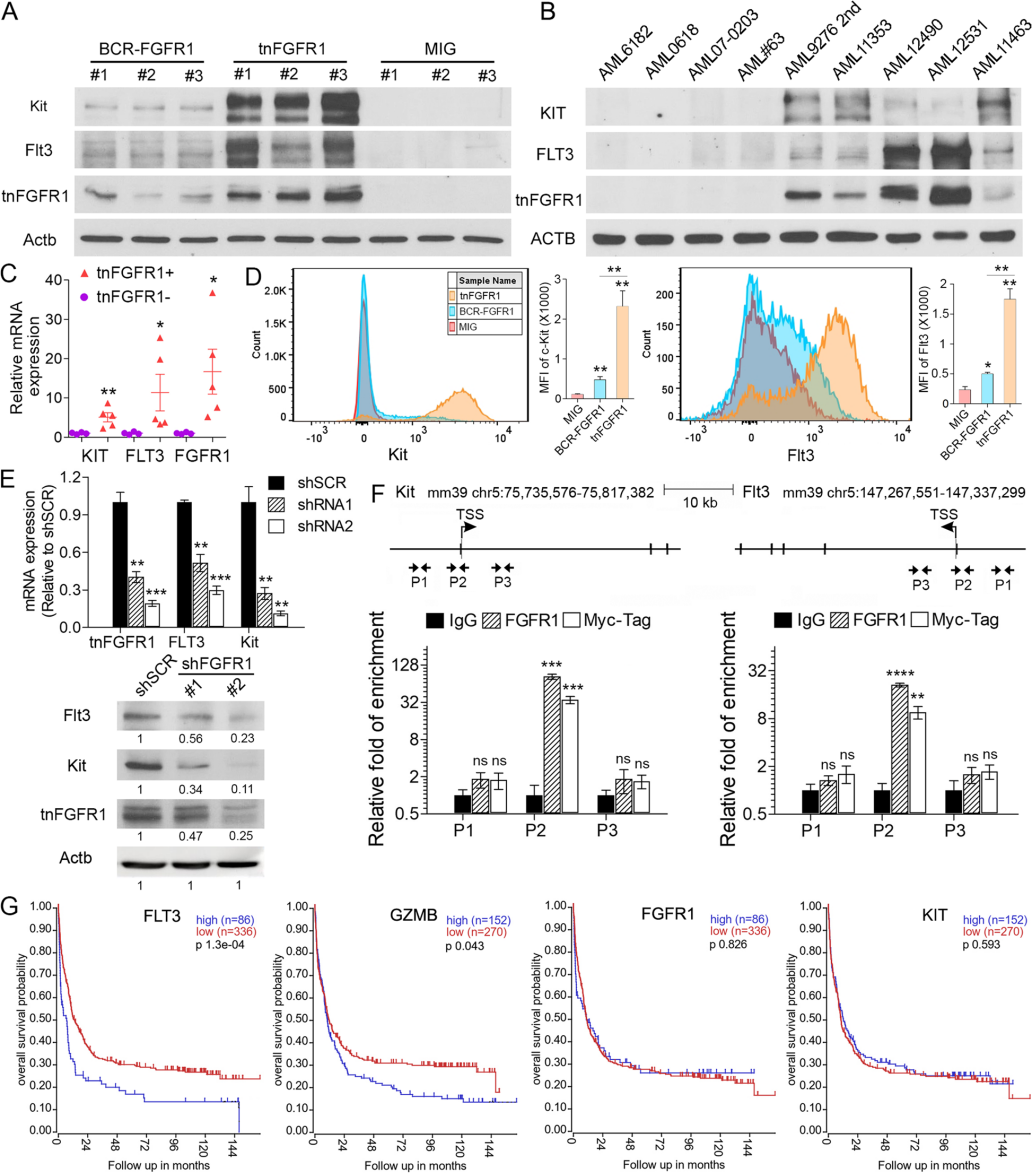
tnFGFR1 activates KIT and FLT3 in primary leukemia cells. Western blot analysis of KIT and FLT3 expression in leukemic cells from primary, tnFGFR1 transduced stem cells shows upregulation of both genes, compared to those cells transduced with BCR-FGFR1m (**A**). A similar correlation was seen in primary human AML cells that either express or do not express tnFGFR1 (**B**). (**C**) qRT-PCR detection of KIT, FLT3 and FGFR1 mRNA expression for human AML samples used for western blotting in (**B**) shows increased levels for all three genes in tnFGFR1 expressing cells. The preferential activation of KIT and FLT3 is further validated by flow cytometry analysis of primary leukemia cell from mouse models (**D**). Using two different shRNAs targeting tnFGFR1 (**E**, above) in primary tnFGFR1 transformed bone marrow cells, qPCR analysis (*N* = 3) shows extensive knockdown of tnFGFR1, Flt3, and Kit mRNA compared with cells transduced with a scrambled shRNA (SCR). Western blot analysis of the same cells (**E**, below) shows effective reduction in FLT3 and KIT protein levels in cells treated with either shRNA. ChIP analysis in primary tnFGFR1 transformed cells isolated from mouse bone marrow, using primers P1 from an upstream intergenic region, P2 within the Kit (**F**, left) or Flt3 (**F**, right) promoters and P3 within intronic regions of the genes, shows enrichment only for the P2 defined promoter regions (*N* = 3). ChIP was performed using antibodies that are specific to tnFGFR1 and also to the MYC-tag that was included in the transfection construct, both of which show the same result. ChIP using IgG alone was performed as a background control. Kaplan-Meier analysis of an AML cohort (*N* = 422, GSE37642) demonstrates a highly significant decrease in overall survival in patients with high expression levels of both FLT3 and GZMB compared with those showing low-level expression. No significant change in survival was found for different levels of expression of FGFR1 or KIT (**G**). ns = not significant. *p ≤ 0.05, **p ≤ 0.01, ***p ≤ 0.001

Significantly, Flt3 and Kit were among the highly upregulated genes in tnFGFR1 expressing cells, and both are known to be involved in maintaining stemness in hematopoietic-stem/progenitor cells (Fig. 5C and D). Western blot analysis of murine stem cells transformed with the full-length BCR-FGFR1 kinase showed relatively low levels of Flt3 and Kit. In the tnFGFR1 transformed cells, however, expression levels of Flt3 and Kit were highly upregulated (Fig. 6A). The relative expression levels in these two cell types was paralleled by expression levels of tnFGFR1. Analysis of a subset of primary human AML samples (Fig. 6B), including four which did not express tnFGFR1 and five that did, showed the same correlation between tnFGFR1 expression and both FLT3 and KIT expression. The significantly upregulated expression of KIT and FLT3 in AML samples exclusively expressing tnFGFR1was also confirmed at the mRNA level using qRT-PCR (Fig. 6C). The correlated expression of tnFGFT1 with Kit and Flt3 were confirmed using flow cytometry of primary leukemia cells from the mouse models (Fig. 6D). Thus, it appears that tnFGFR1, directly or indirectly, influences FLT3 and KIT expression. For most of the archival AML samples described in Fig. 3B, clinical annotation details were not available but in the cases shown in Fig. 6B, AML sample 11,463 was positive for the Flt3 ITD mutation while samples #9276, 11,353 and 12,490, although tested did not show the ITD mutation.

To explore the relationship between tnFGFR1 and FLT3/KIT expression, we designed shRNAs that targeted tnFGFR1 and transfected them into tnFGFR1 transformed primary leukemic cells isolated from bone marrow of mice receiving the transduced cells. As shown in Fig. 6E, as a result of targeting with two shRNAs, which effectively reduced tnFGFR1 expression levels (Fig. 6E, upper) and protein levels (Fig. 6E, lower), there was a significant reduction in the expression of both Flt3 and Kit compared with cells transduced with virus containing a scrambled shRNA. To determine whether this was a direct transcriptional regulation, we performed ChIP analysis of the Flt3 and Kit promoters in primary tnFGFR1 transformed cells (Fig. 6F). In these experiments the transfected tnFGFR1 gene carries a MYC-tag allowing a specific analysis of its expression. Three primer pairs were used to analyse tnFGFR1 occupancy on these promoters, one (P1) upstream in an intergenic region, another within the Flt3/Kit promoters (P2) and a third (P3) located within an intronic region but beyond the promoter region. In both cases, high levels of tnFGFR1 were recovered by ChIP using the P2 primers that were specific for pull-downs using either the tnFGFR1 antibody or the Myc tag antibody. These results demonstrate the selective occupancy of tnFGFR1 on the promoters of these two genes. Thus, it appears that activation of Flt3 and Kit in this cell system is a direct result of tnFGFR1 acting in a transcriptional capacity.

In gene expression data derived from primary human AML samples (GSE37642), while there was no difference in overall survival between patients in which FGFR1 was expressed at high or low levels, there was a significant difference depending on expression levels of FLT3 (Fig. 6G). On the other hand no difference in survival was seen relative to KIT expression levels. Interestingly, high-level expression of GZMB was also associated with poor overall survival. This inferior prognosis associated with high GZMB and FLT3 expression levels, but not with FGFR1 or KIT, was further confirmed in the independent AML patient samples from the TCGA panCancer Atlas (Supplement Figure 5). Here, however, overexpression of FGFR1 and KIT was associated with better survival. While the reason for the survival difference between high and low expression of these genes in this TCGA dataset and the lack of a significant difference in GSE37642 dataset is not clear, it appears that expression of these two genes may not be a valuable predictor for better survival. In both AML patient cohorts, however, higher GZMB expression alone can be used as an independent indicator for poor disease outcome. Given that tnFGFR1 is produced by GZMB cleavage, and is involved in FLT3 activation, these patient survival data may suggest an important, but as yet underappreciated role of tnFGFR1 in human AML.

### Targeting FLT3 as a treatment for tnFGFR1 driven leukemia

**Fig. 7:**
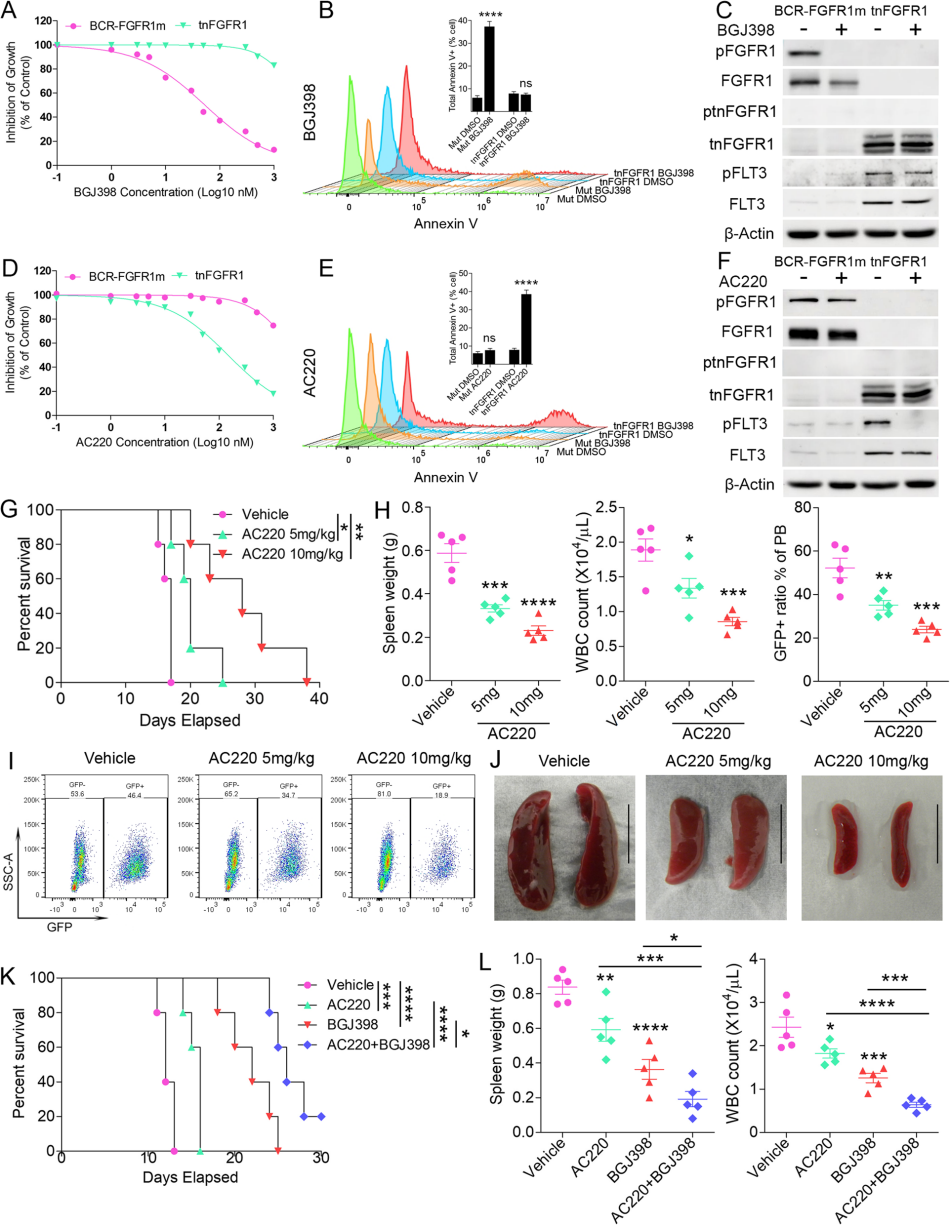
Targeting Flt3 can inhibit leukemia progression driven by tnFGFR1. When the primary cells from mice xenografted with BCR-FGFR1m or tnFGFR1 were treated with increasing concentrations of BGJ398 (*N* = 3), the BCR-FGFR1m cells show a dose dependent suppression of growth whereas the tnFGFR1 transformed cells only show a mild suppression of growth at the highest concentrations (**A**). Flow cytometric analysis of Annexin V staining (**B**) shows significant apoptosis only in the BCR-FGFR1m expressing cells. BGJ398 treatment specifically blocks phosphorylation of the full length fusion kinase, but has no effect on the tnFGFR1 and FLT3 signaling (**C**). When the same cells were treated with the AC220 FLT3 inhibitor (**D**), the tnFGFR1 transduced cells show a dose dependent suppression of growth, with the BCR-FGFR1m cells only showing slight growth suppression at the highest concentrations. Increased Annexin V staining in this case was only seen in the tnFGFR1 cells treated with AC220 (**E**). AC220 treatment specifically targets the Flt3 signaling (**F**). Survival analysis of mice (*N* = 5) xenografted with tnFGFR1 expressing cells shows a dose-dependent improvement in mouse survival following treatment with AC220, which is reflected in spleen weight and WBC count as well as the percentage of GFP+ leukemia cells in the peripheral blood (**G**-**J**). When mice (*n* = 5) inoculated with primary BCR-FGFR1 expressing leukemia cells and then treated after 7 days with either BGJ398 (30 mg/Kg) or AC220 (10 mg/Kg) alone, ANOVA statistical analysis with Bonferroni correction revealed that there was a significant increase in survival when each cohort was compared individually with the vehicle treated control group (**K**). A combination of BGJ398 and AC220 shows a further significant improvement in survival compared with single treatment of the two drugs. In pairwise comparisons the changes in survival were reflected in the spleen weight and the WBC between various treatments (**L**). ns = not significant. *p ≤ 0.05, **p ≤ 0.01, ***p ≤ 0.001, ****p ≤ 0.0001. Bonferroni-Adjusted Alpha is 0.0125 for the combinational drug treatment

In previous studies we have demonstrated that SCLL cells, both in vitro and in vivo, are remarkably sensitive to FGFR1 inhibitors whose major function is to prevent the phosphoactivation of the kinase [10]. Among the various FGFR1 inhibitors, BGJ398 has proved to be the most effective drug [10] and in the BaF3 cell system, while it was particularly effective in suppressing growth in both BCR-FGFR1 and BCR-FGFR1m kinases in a dose dependent manner, tnFGFR1 expressing cells were relatively insensitive (Supplement Figure 6A and B). This suppression of growth was also observed in primary bone marrow cells expressing BCR-FGFR1m which was associated with an increase in Annexin V staining as determined by flow cytometry (Fig. 7A and B) as well as progression through the cell cycle (Supplement Figure 6C and D). Western blot analysis demonstrates treatment with BGJ398 suppresses activation of BCR-FGFR1m but has no effect on tnFGFR1 or FLT3 activation (Fig. 7C). Cells expressing tnFGFR1 are resistant to FGFR1 inhibitors but, because FLT3 is upregulated in these cells and not in the mutant kinase expressing cells, when treated with the AC220 [31] FLT3 inhibitor (Fig. 7D), the tnFGFR1 expressing cells showed a dose dependent suppression of growth, whereas the mutant kinase expressing cells were relatively insensitive. This suppression in growth is also reflected in the delayed progression through the cell cycle (Supplement Figure 6C and D). Analysis of apoptosis in these cells showed increased levels of Annexin V in the tnFGFR1 cells treated with AC220 (Fig. 7E). When these cells are treated with AC220, activation of BCR-FGFR1m is unaffected but there is a reduction in FLT3 activation in tnFGFR1 expressing cells (Fig. 7F). To extend these studies to in vivo models, tnFGFR1-expressing leukemia cells were serially xenografted into BALB/C hosts and allowed to engraft for 7 days. Starting on day 8, individual mice were then treated daily with either 5 mg/Kg or 10 mg/Kg of AC220 until death of the first mouse in the control group (Day 15) when the treatment was terminated. There was a dose dependent improvement of cell survival time proportional to the dose of AC220, compared with mice treated with vehicle alone (Fig. 7G). which was reflected in spleen weight, white blood cell count and proportion of WBC expressing GFP (Fig. 7H-J), indicating the dependence of these tnFGFR1 expressing leukemia cells on FLT3 and providing a possible targeting strategy for this leukemia.

Considering the FGFR1 related leukemia are driven by both the full-length fusion kinase and the truncated tnFGFR1, we examined the long-term efficacy of the combination of FLT3 and FGFR1 inhibitors with our SCLL leukemia model. Recipient mice engrafted with primary leukemia cell expressing wild type BCR-FGFR1 were treated with FGFR1 and FLT3 inhibitor alone or in combination. When AC220 (10 mg/Kg) was used in combination with the BGJ398 FGFR1 inhibitor (30 mg/Kg) there was not only a highly significant increase in survival compared with AC220 treatment alone but also a significant increase in survival compared with BGJ398 treatment alone (Fig. 7K). Again, the responses to the different drugs and combination of drugs was reflected in the spleen weights and WBC at the time of autopsy (Fig. 7L). This superior survival further underscores the therapeutic importance of our original discovery of the leukemia promoting role by the non-classical truncated nuclear FGFR1 variant and provides a potentially improved treatment of all FGFR1 related leukemias.

## Discussion

The consistent observation that chromosome abnormalities involving 8p11 in SCLL, often as the only cytogenetic change [32], pointed to the importance of the chimeric kinases in the overall transformation process [4]. Because of the acquired dimerization motifs from the fusion partner, the chimeric kinase is constitutively phosphoactivated. As a consequence, it has been suggested that their ability to transform hematopoietic stem cells results from activation of endogenous, oncogenic proteins such as STAT3/5, SRC [5] and PLCG, for example, some of which are also activated by the ligand-dependent, normal FGFR1 during hematopoiesis. Indeed we have demonstrated that the serine-threonine kinase domain in the BCR component of the fusion kinase, for example, can promote more aggressive leukemogenesis through associated interactions with SHP2 [6]. The demonstration here that the BCR-FGFR1m chimeric kinase efficiently transforms HSCs, points to the importance of protein activation events in the cytoplasm in the transformation process. The endogenous oncogenic capacity of the truncated FGFR1 derivative, which is composed only of the tyrosine kinase domain and localizes exclusively to the nucleus, points to critical oncogenic nuclear events. Transformation by the wild type chimeric kinases, therefore, is presumably due to a combined effect of the activation of endogenous proteins as well as through the action of tnFGFR1 in the nucleus. Gene expression data support this suggestion, since the fusion kinases and tnFGFR1 have different and distinct influences on reorganization of the gene expression profile in SCLL cells. The BCR-FGFR1m derivative, which cannot generate tnFGFR1, and which is exclusively located in the cytoplasm [17], largely shows the same disease profile as the parent fusion kinase in terms of immunophenotype and progression characteristics, although consistently has a slightly longer latency period in vivo. It is possible this may be due to the absence of gene function directed by tnFGFR1 in these cells. Gene expression profiling of cells transformed with these two oncogenes, however, implies that the cytoplasmic BCR-FGFR1 variant affects fundamental mechanisms related to cell growth and transformation, while the tnFGFR1 derivative has a different influence on genes that are related to maintaining the undifferentiated stem cell state. Nonetheless, tnFGFR1 can independently lead to transformation of stem cells, possibly as a result of maintaining a stem cell phenotype, providing a pool of undifferentiated cells with proliferative potential in which the subsequent acquisition of oncogenic gene mutations can promote the progression of the disease. This may account for the observations in the BaF3 and primary HSC cell systems where, unlike the chimeric kinases, tnFGFR1 was not able to drive acutely transformation of hematopoietic cells.

The suggestion of two independent mechanisms of action ascribed to the chimeric FGFR1 kinases raises important questions about the use of FGFR1 inhibitors in the treatment of this disease. FGFR1 inhibitors largely target the ATP-binding sites in the kinase domain preventing activation [33] but, since the action of tnFGFR1 in the nucleus does not depend on its phosphorylation, its function is not suppressed. Thus, while FGFR1 inhibitors appear to be effective in the first instance in SCLL models, when the chimeric kinases are the dominant species of the protein, the fact that the tnFGFR1 protein can become the dominant species over time, possibly as a result of increased GZMB activity, may require alternative strategies to target these leukemia cells.

A role for the truncated FGFR1 as a transcription factor was suggested from analysis of Myc activation in SCLL [17], where ChIP analysis suggested occupancy on the Myc promoter. However since truncated FGFR1 does not have a DNA binding motif this is presumed to function through association with, as yet unidentified, proteins. A role for normal FGFR1 in the nucleus has been described [34], although without distinction between the full length and truncated forms, which affects normal cell maturation and differentiation of neuronal cells through a variety of pathways [35] facilitated by association with proteins such as the CREB binding factor (CBF). The description of a truncated FGFR1 (also referred to as nFGFR1) as a result of Granzyme B, was described in breast cancer cells and claimed to affect gene expression that altered cell behavior such as invasion [16]. To make the distinction between different nuclear forms we have suggested using the terminology tnFGFR1 for the truncated, nuclear derivative. In our studies, this truncated tnFGFR1 appears to maintain a stem cell phenotype in hematopoietic stem/precursor cells by regulating expression of genes such as Flt3 and Kit. Clearly, the generation of truncated FGFR1 depends on the expression of Granzyme B which is highly upregulated in hematopoietic cell lineages [36]. The SCLL model we describe here, therefore, offers an ideal opportunity to study the effects of truncated FGFR1-directed gene expression promoting malignant progression in HSCs.

Gene expression profiling in tnFGFR1 expressing cells highlighted upregulation of the FLT3 and KIT genes. KIT is expressed in a subset of hematopoietic stem cells, including CD34+ cells, and has been reported in ~ 70% of AML [37–41]. It is also essential for normal hematopoiesis and is downregulated during HSC cell maturation. The high levels of KIT in the tnFGFR1 transformed cells further supports the idea that the truncated kinase promotes stemness in these cells. FLT3 is a transcription factor that plays a normal role in the development and differentiation of hematopoietic stem cells through regulation of genes involved in these normal processes [42]. FLT3 has also been extensively studied in the development of AML, where the focus has been on the role of the various mutations in enhancing disease progression and survival [43, 44]. It should be noted, however, that upregulation of wild type FLT3 is also a common feature of AML and since tnFGFR1 has a direct effect on the Flt3 promoter, upregulation of FLT3 in all its forms would be predicted. What was surprising, however, was that in the limited set of AML that were available to us, it appears that there was an exclusive association between expression of tnFGFR1 and FLT3.

In the comparison of gene expression between cells expressing the mutant FGFR1 (which does not generate tnFGFR1) and tnFGFR1-expressing cells, there were 952 gene showing significant, > 4-fold, differences in expression levels. These genes are candidates for direct or indirect, regulation by tnFGFR1. Within this group, we focused on genes involved in three specific categories that have relevance to the hypothesis that transformation by tnFGFR1 facilitates regulation of genes promoting leukemogenesis while retaining stemness. There were relatively few traditional oncogenes (p53, BRCA1 etc.) showing gene expression changes and only five genes in this group showed significant expression changes. Among these were the Bmyc and MycL members of the MYC oncogene family. Significantly, the MYB oncogene was upregulated, which is consistent with our previous observations of its FGFR1-dependent upregulation in SCLL models [17].

Observations using flow cytometry demonstrated that while the mutant FGFR1gives rise to leukemia with a pre-B cell phenotype, cells transformed by tnFGFR1 exhibit a more stem cell like immunophenotype. Overall nine genes which promote stemness were overexpressed and are candidates to explain this phenomenon. Included in this sub-group are the Flt3 and Kit genes which are discussed above. In addition, genes such as Angptl1, Tmem119 and Gfra1 are involved in stem cell maintenance [45–47] and Ccl9 and Met are related to stem cell differentiation [48, 49].

From our previous work, tnFGFR1appears to act as a transcription factor to promote expression of genes involved in the leukemia phenotype in HSCs [17]. However, since tnFGFR1 cannot bind DNA directly, it likely exerts it influence on gene expression through association with other transcription factors/cofactors. While confirmation of these interactions will require am extensive proteomics approach, some insights come from the gene expression studies. In the genes with > 4 fold increased expression, 40 had a functional annotation as transcription factors/cofactors. Within this diverse group are LMO1 and LMO2 which have a central role in leukemogenesis [50]. Several of the genes highlighted in Supplement Table 1 also act as transcription cofactors and transcription repressors which are candidates for the contributing to tnFGFR1 transformation through regulation of gene expression. Of those genes implicated in stemness include Klf3, Klf4, Six1 and Hoxb7. Of particular interest is the upregulation of Hopx which is implicated in promoting stemness in leukemic stem cells in AML [51]. While upregulation of these genes cannot be assigned to interacting with tnFGFR1, our previous studies with the Myc oncogene demonstrate that a positive feedback loop of expression and consequent function has a key role in the transformation process of HSCs by FGFR1 kinase.

Analysis of primary human AML in this study was limited to archival materials for which clinical annotation was largely missing, precluding a definitive association between the presence of mutant FLT3 and tnFGFR1. From the limited data available, however, it appears that increases in FLT3 expression is seen for both the ITD mutation as well as apparently normal FLT3, although point mutations that occur throughout the gene cannot be excluded. These observations lay the platform for prospective studies to establish the relationship between expression of the mutant and wild type FLT3 and tnFGFR1 in transformed stem cells. The prediction, since tnFGFR1 appears to bind the FLT3 promoter, is that expression of all FLT3 variants will be upregulated in the presence of tnFGFR1.

The observation in human AML that tnFGFR1 can apparently exist in the absence of the full length kinase is presumably an artifact of the western blotting detection system, since tnFGFR1 is generated by enzymatic cleavage of the parental kinases. It is possible that low-level expression of the full length FGFR1 together with a rapid GZMB cleavage may give the appearance of exclusive expression of tnFGFR1. We present evidence that the tnFGFR1 derivative is not generated as a result of DNA modification or alternative mRNA splicing but occurs as a post-translational event. The demonstration that GZMB levels correlate with tnFGFR1 levels support this explanation. Gene expression analysis shows that GZMB levels are increased in tnFGFR1 expressing cells, suggesting an autocrine activation loop resulting in a more efficient processing of the full-length proteins, thereby diminishing their presence in the cells. This increase in tnFGFR1 as tumors progress accentuates the increase in tnFGFR1 regulated genes such as FLT3, which is well established to promote aggressive progression of AML [43, 44]. The apparent dependence on tnFGFR1 for FLT3 expression and the requirement for GZMB to generate tnFGFR1 possibly suggests a mechanism behind suppression of AML development as a result of targeting GZMB.

The observation that tnFGFR1 can be a dominant driver of AML development that leads to upregulation of FLT3 has important implications in targeted therapies for this distinct subgroup of AML. In a limited preclinical study [10], we have shown that leukemias with upregulation of FGFR1 are sensitive to a wide variety of FGFR1 inhibitors. As the disease progresses, however, apparently as a result of increased GZMB expression, tnFGFR1 becomes dominant and renders the cells insensitive to FGFR1 inhibitors. These cells, however, show upregulation of FLT3 and are sensitive to AC220. In preclinical studies using a combination of FLT3 and FGFR1 inhibitors an improved overall survival was noted, suggesting that a combination of FGFR1 and FLT3 inhibitors at an early stage may provide a more effective strategy.

## Conclusions

The truncated FGFR1 kinase (tnFGFR1) can independently transform murine hematopoietic stem cells through molecular mechanisms that are distinct from those seen for the chimeric kinases characteristic of SCLL. Primary human de novo AML also show the presence of tnFGFR1 in up to 50% of cases analysed. In these cells a correlation was seen between tnFGFR1 and the upregulation of the stem cell determining genes FLT3 and KIT, which appears to be due to the action of tnFGFR1 at the promoters of these genes. These observations have important implications for the treatment of AML expressing the FGFR1 kinases since the transforming capacity of tnFGFR1 makes them insensitive to FGFR1 inhibitors. Given the correlation between tnFGFR1 and FLT3 expression, however, application of FLT3 inhibitors can potentially overcome this resistance and provide a novel treatment option for related leukemias.

## Supplementary Information

**Additional file 1: Supplement Figure 1.** H&E staining analysis of spleen sections from mice xenografted with cells expressing each of the FGFR1-derivative constructs demonstrates disruption of the normal follicular structure seen in the mice receiving cells with the MIG expression vector alone, supporting the leukemia diagnosis.

**Additional file 2: Supplement Figure 2.** Representative flow cytometry analysis of GFP positive cells from the spleens of mice (*N* = 5) transduced with BCR-FGFR1, BCR-FGFR1m and tnFGFR1 compared with the empty MIG vector (GFP negative cells). In all cases the leukemic cells are B220+ CD4-CD8-Gr1-. Cells transduced with tnFGFR1 show a small percentage (12.6%) of Mac1 + Gr1+ cells but unlike the other cell populations are almost exclusively Kit+Sca1+ (A). Scatter plots from analysis of each individual mouse in the different cohorts are shown in (B). ns = not significant. *p ≤ 0.05, ***p ≤ 0.001, ****p ≤ 0.0001.. Each comparison represents a pairwise analysis between the individual oncogenic kinase cohorts and the MIG empty vector control group.

**Additional file 3: Supplement Figure 3.** Western blot analysis of an additional nine AML samples with AML1049 and AML#40 showing weak but exclusive tnFGFR1 expression (A). qRT-PCR analysis shows equal enrichment of transcripts from the 3′- and 5′- ends of FGFR1 mRNA following continuous transplantation in PDX models, with a significant upregulation of GZMB in AML9276 compared to AML#10. ns = not significant. **p ≤ 0.01.

**Additional file 4: Supplement Figure 4.** RNA-Seq data analysis from two independent experiments for BCR-FGFR1m and tnFGFR1 expressing cells compared with flow sorted, normal Lin-Kit+ hematopoietic stem cells reveals distinct groups of genes expressed specifically in the various subtypes of cells (A). In a comparison of genes expressed in the BCR-FGFR1m that are not expressed in the normal stem cells or tnFGFR1 expressing cells (indicated by the orange bar to the left), the 10 most significant GO categories are all related to rapid cell growth and proliferation (B, upper). In contrast, analysis of the genes that are specifically expressed in the tnFGFR1 transformed cells (indicated by the pink bar to the left) the top 10 most significant GO categories are largely related to functions involved with cell differentiation (B, below).

**Additional file 5: Supplement Figure 5.** Kaplan-Meier analysis of AML samples from the TCGA PanCancer Atlas, demonstrates high GZMB and FLT3 expression are correlated with poor prognosis, while high FGFR1 and KIT expression are associated with a superior disease outcome.

**Additional file 6: Supplement Figure 6.** When treated with increasing concentrations of BGJ398, BaF3 cells (*N* = 3) transduced with wild-type BCR-FGFR1 and BCR-FGFR1m cells show a dose dependent suppression of growth after 3 days, whereas the tnFGFR1 transformed cells only show a mild suppression of growth at the highest concentrations (A, B). Flow cytometric analysis (C) of primary bone marrow cells isolated from mice inoculated with either BCR-FGFR1m or tnFGFR1 was used to assess the percentage of cells in various stages of the cell cycle (D). After 18 hours treatment, cells expressing BCR-FGFR1m show increased levels of cells in G0/G1 phase arrest when treated with BGJ398 but there is no effect compared with cells treated with DMSO when the same cells are treated with AC220. In contrast, in cells expressing tnFGFR1, BGJ398 has no effect on cell cycle progression whereas AC220 results in an increase in cells in the G0/G1 phase of the cell cycle (D).

**Additional file 7: Supplement Table 1.** Summary of gene expression changes in tnFGFR1 expressing cells compared with cells expressing BCR-FGFR1m in the categories of ‘Oncogene’, transcription factor’ and involved in maintaining ‘stemness’. Gene expression (GE) is cited as the mean FPMK values (N-2) in both cases.

## Data Availability

The RNA-Seq data reported in this article has been deposited under the gene expression omnibus (GEO) accession number GSE.

## Abbreviations

FGFR1: Fibroblast growth factor receptor 1
SCLL: Stem cell leukemia lymphoma syndrome
FLT3: Fms-like tyrosine kinase 3
AML: Acute myelogenous leukemia
GZMB: Granzyme B
IL3: Interleukin 3
BM: Bone marrow
GFP: Green fluorescent protein
qRT-PCR: Quantitative reverse polymerase chain reaction
RPPA: Reverse phase protein array
PDX: Patient derived xenografts
GO: Gene ontology
KIT: KIT proto-oncogene
BCR: Breakpoint cluster region
GSEA: Gene set enrichment analysis
WBC: White blood cell
FPMK: Fragments per million kilobases
