# Supplementary figures and images for: A truncated derivative of FGFR1 kinase cooperates with FLT3 and KIT to transform hematopoietic stem cells in syndromic and de novo AML
Source: Mol Cancer. 2022 Jul 29;21:156. doi: 10.1186/s12943-022-01628-3 (PMC9336057; doi:10.1186/s12943-022-01628-3)

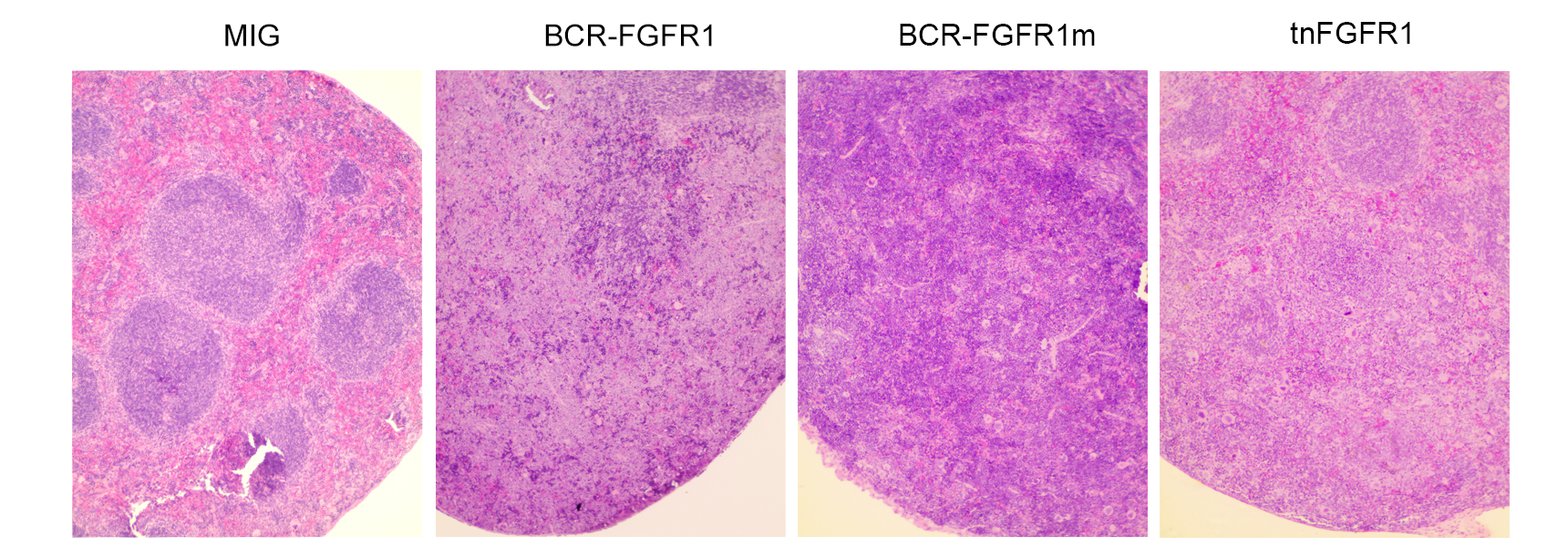

Supplement: Supplementary file 1 — Additional file 1: Supplement Figure 1. H&E staining analysis of spleen sections from mice xenografted with cells expressing each of the FGFR1-derivative constructs demonstrates disruption of the normal follicular structure seen in the mice receiving cells with the MIG expression vector alone, supporting the leukemia diagnosis. [file 12943_2022_1628_MOESM1_ESM.tif]

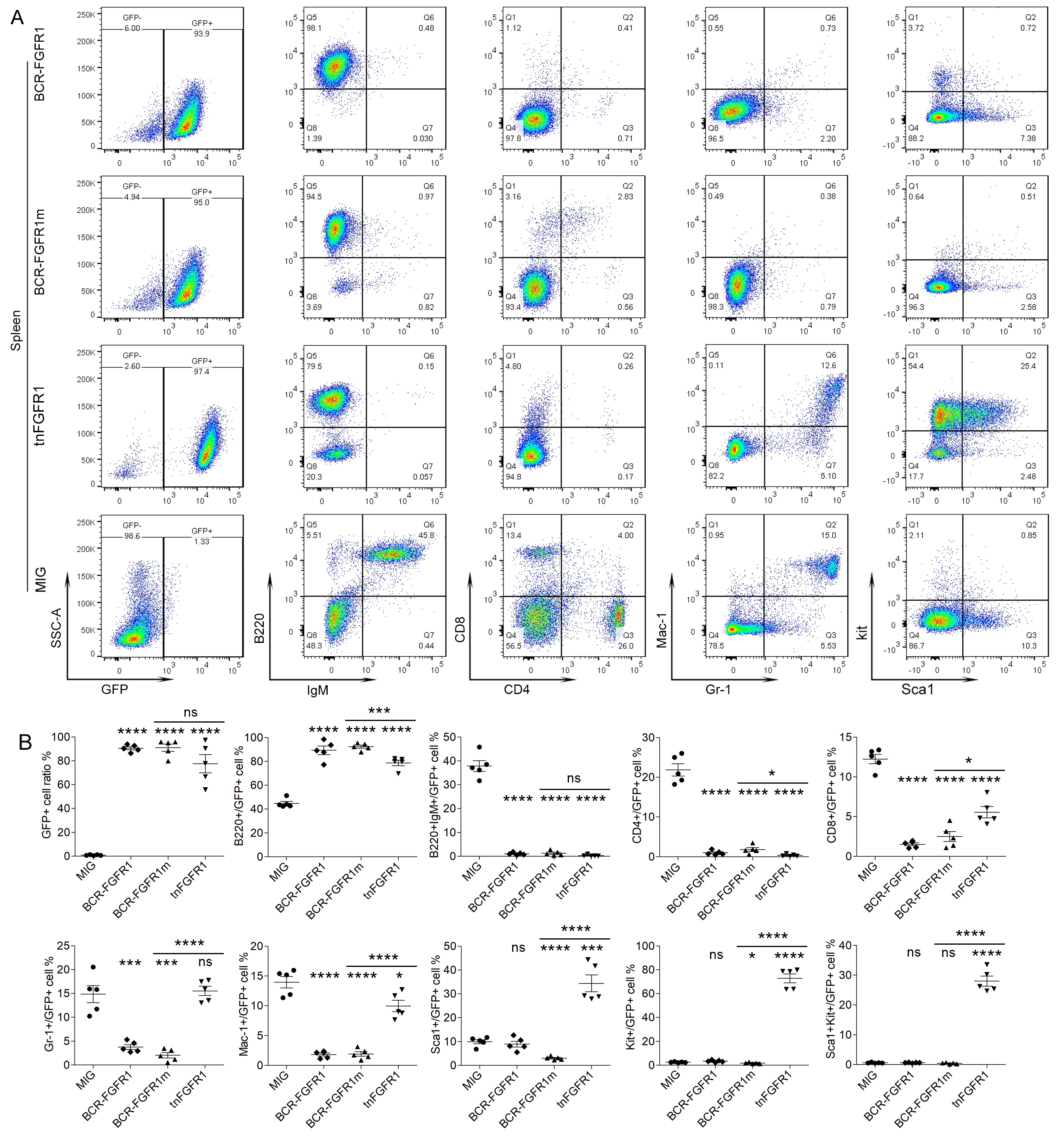

Supplement: Supplementary file 2 — Additional file 2: Supplement Figure 2. Representative flow cytometry analysis of GFP positive cells from the spleens of mice (N = 5) transduced with BCR-FGFR1, BCR-FGFR1m and tnFGFR1 compared with the empty MIG vector (GFP negative cells). In all cases the leukemic cells are B220+ CD4-CD8-Gr1-. Cells transduced with tnFGFR1 show a small percentage (12.6%) of Mac1 + Gr1+ cells but unlike the other cell populations are almost exclusively Kit+Sca1+ (A). Scatter plots from analysis of each individual mouse in the different cohorts are shown in (B). ns = not significant. *p ≤ 0.05, ***p ≤ 0.001, ****p ≤ 0.0001.. Each comparison represents a pairwise analysis between the individual oncogenic kinase cohorts and the MIG empty vector control group. [file 12943_2022_1628_MOESM2_ESM.tif]

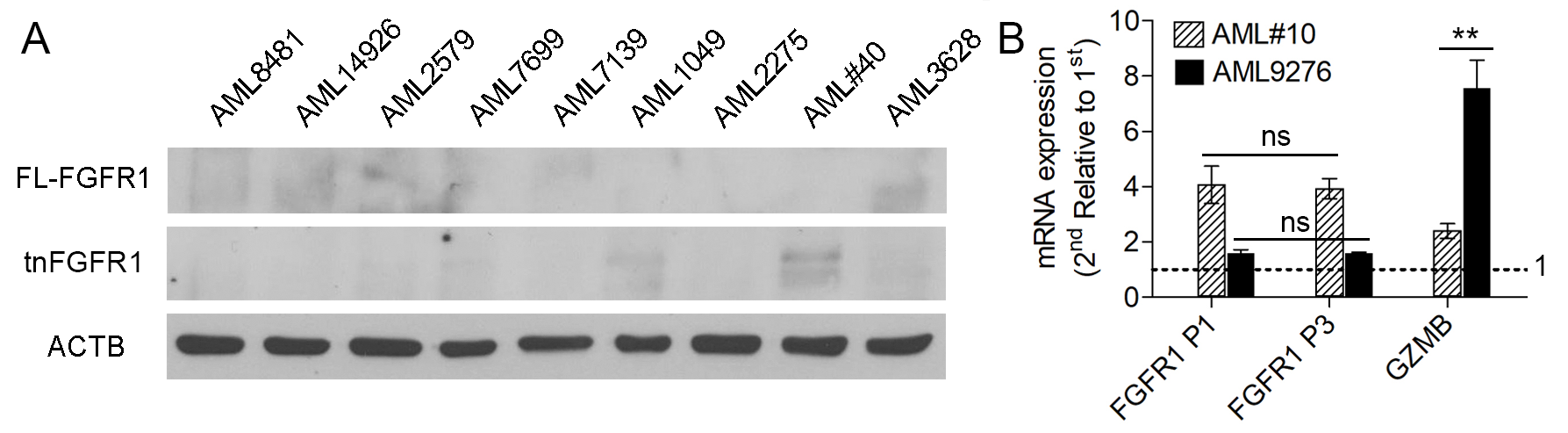

Supplement: Supplementary file 3 — Additional file 3: Supplement Figure 3. Western blot analysis of an additional nine AML samples with AML1049 and AML#40 showing weak but exclusive tnFGFR1 expression (A). qRT-PCR analysis shows equal enrichment of transcripts from the 3′- and 5′- ends of FGFR1 mRNA following continuous transplantation in PDX models, with a significant upregulation of GZMB in AML9276 compared to AML#10. ns = not significant. **p ≤ 0.01. [file 12943_2022_1628_MOESM3_ESM.tif]

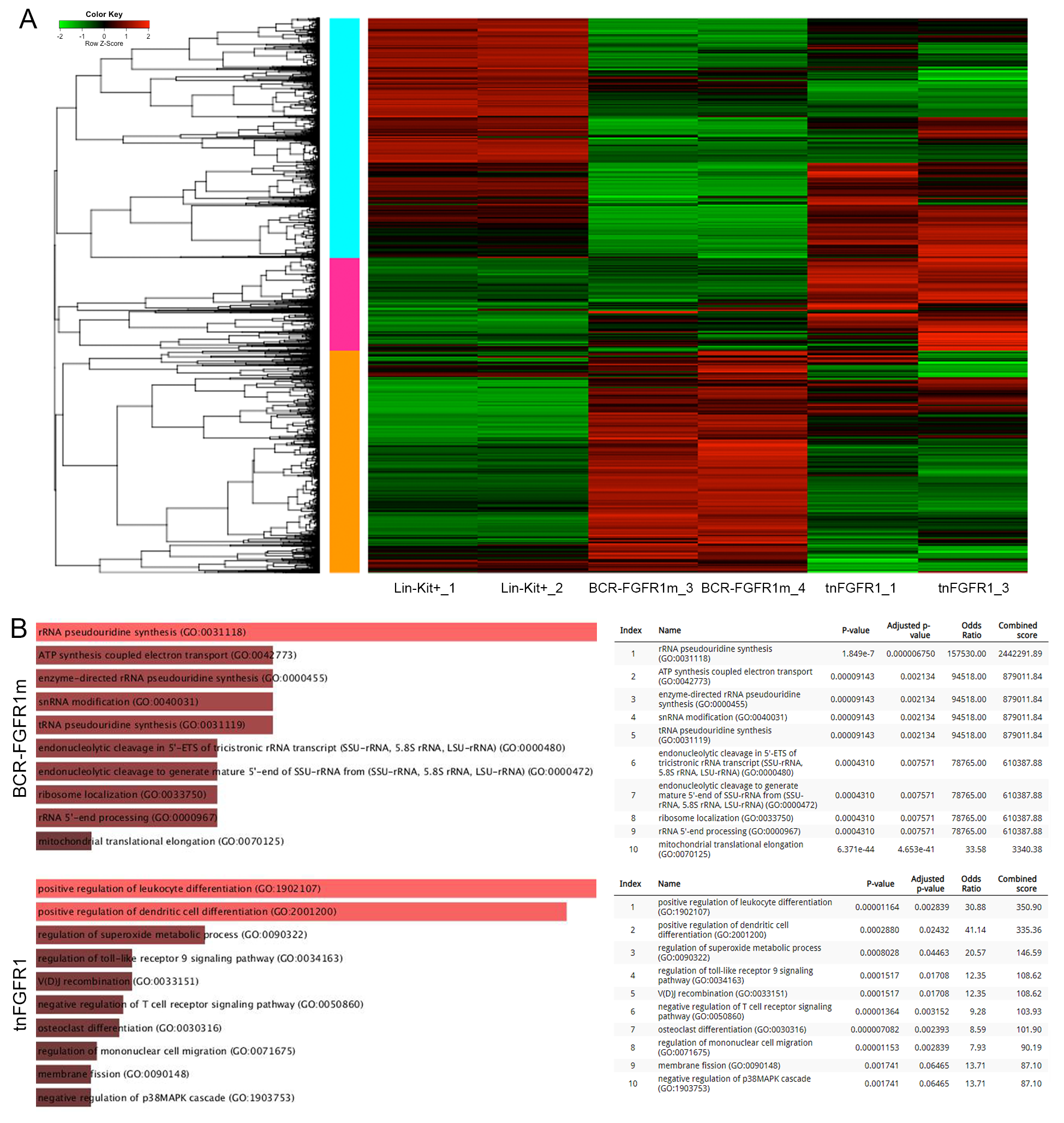

Supplement: Supplementary file 4 — Additional file 4: Supplement Figure 4. RNA-Seq data analysis from two independent experiments for BCR-FGFR1m and tnFGFR1 expressing cells compared with flow sorted, normal Lin-Kit+ hematopoietic stem cells reveals distinct groups of genes expressed specifically in the various subtypes of cells (A). In a comparison of genes expressed in the BCR-FGFR1m that are not expressed in the normal stem cells or tnFGFR1 expressing cells (indicated by the orange bar to the left), the 10 most significant GO categories are all related to rapid cell growth and proliferation (B, upper). In contrast, analysis of the genes that are specifically expressed in the tnFGFR1 transformed cells (indicated by the pink bar to the left) the top 10 most significant GO categories are largely related to functions involved with cell differentiation (B, below). [file 12943_2022_1628_MOESM4_ESM.tif]

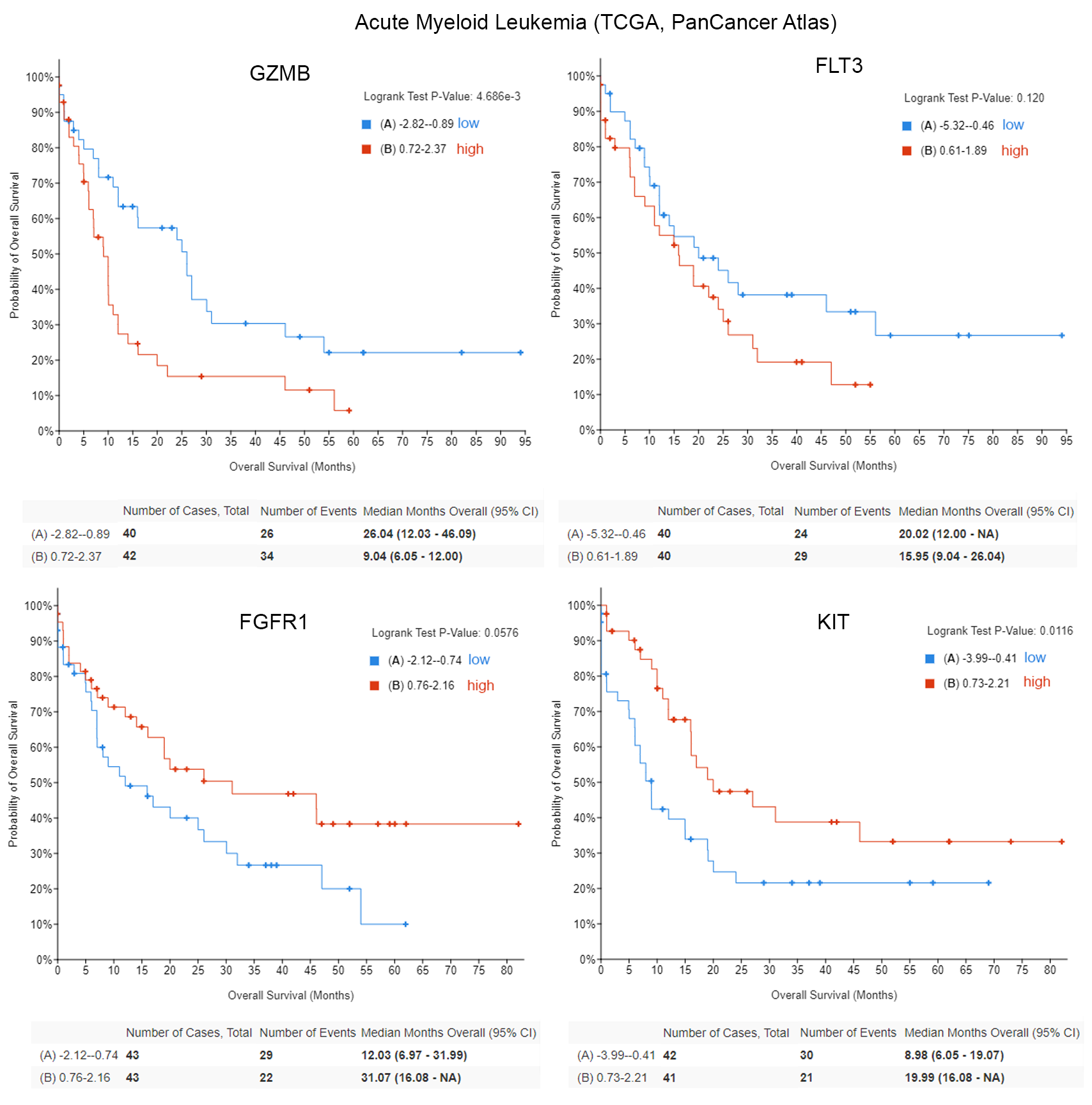

Supplement: Supplementary file 5 — Additional file 5: Supplement Figure 5. Kaplan-Meier analysis of AML samples from the TCGA PanCancer Atlas, demonstrates high GZMB and FLT3 expression are correlated with poor prognosis, while high FGFR1 and KIT expression are associated with a superior disease outcome. [file 12943_2022_1628_MOESM5_ESM.tif]

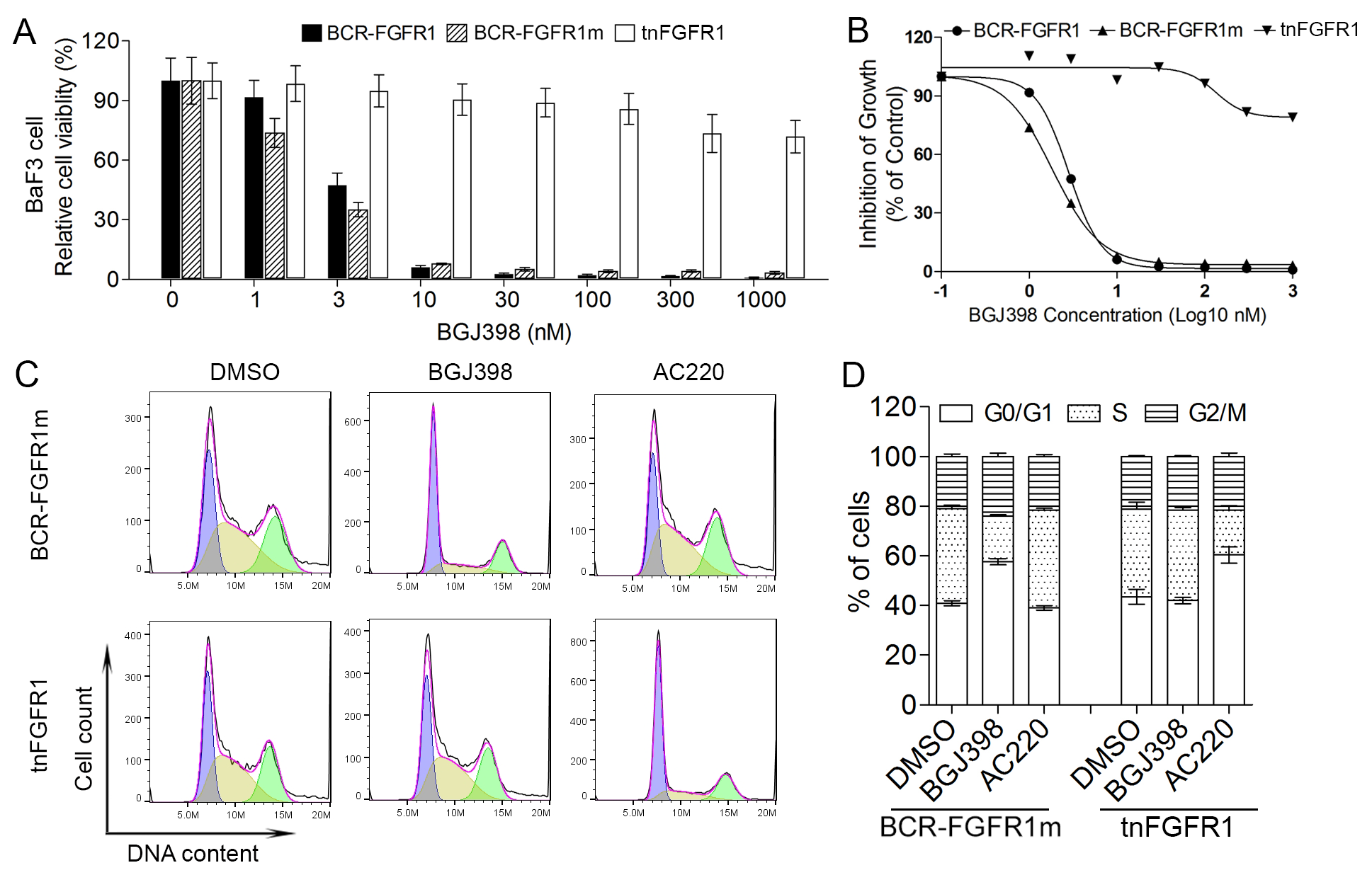

Supplement: Supplementary file 6 — Additional file 6: Supplement Figure 6. When treated with increasing concentrations of BGJ398, BaF3 cells (N = 3) transduced with wild-type BCR-FGFR1 and BCR-FGFR1m cells show a dose dependent suppression of growth after 3 days, whereas the tnFGFR1 transformed cells only show a mild suppression of growth at the highest concentrations (A, B). Flow cytometric analysis (C) of primary bone marrow cells isolated from mice inoculated with either BCR-FGFR1m or tnFGFR1 was used to assess the percentage of cells in various stages of the cell cycle (D). After 18 hours treatment, cells expressing BCR-FGFR1m show increased levels of cells in G0/G1 phase arrest when treated with BGJ398 but there is no effect compared with cells treated with DMSO when the same cells are treated with AC220. In contrast, in cells expressing tnFGFR1, BGJ398 has no effect on cell cycle progression whereas AC220 results in an increase in cells in the G0/G1 phase of the cell cycle (D). [file 12943_2022_1628_MOESM6_ESM.tif]
