# Supplementary material for: A truncated derivative of FGFR1 kinase cooperates with FLT3 and KIT to transform hematopoietic stem cells in syndromic and de novo AML
Source: Mol Cancer. 2022 Jul 29;21:156. doi: 10.1186/s12943-022-01628-3 (PMC9336057; doi:10.1186/s12943-022-01628-3)
Supplement: Supplementary file 7 — Additional file 7: Supplement Table 1. Summary of gene expression changes in tnFGFR1 expressing cells compared with cells expressing BCR-FGFR1m in the categories of ‘Oncogene’, transcription factor’ and involved in maintaining ‘stemness’. Gene expression (GE) is cited as the mean FPMK values (N-2) in both cases. [file 12943_2022_1628_MOESM7_ESM.docx]

|  |  |  |  |  |  |
| --- | --- | --- | --- | --- | --- |
| **Gene** | **GE mut** | **GE nFGFR1** | **fold change** | **p-value** | **q-value** |
|  |  |  |  |  |  |
| **Oncogenes** |  |  |  |  |  |
|  |  |  |  |  |  |
| Bmyc | 0.42139 | 15.0891 | 35.75 | 0.00305 | 0.00760431 |
| Dleu7 | 0.0571057 | 1.21047 | 21.11 | 0.09635 | 0.153843 |
| Lzts1 | 0.207809 | 1.7167 | 8.26 | 0.0008 | 0.00230168 |
| Myb | 32.4533 | 189.102 | 5.816 | 5.00E-05 | 0.00018571 |
| Mycl | 0.761484 | 4.35626 | 5.72 | 5.00E-05 | 0.00018571 |
|  |  |  |  |  |  |
| **Transcription factors** | |  |  |  |  |
|  |  |  |  |  |  |
| Lmo1 | 0.686412 | 46.6365 | 67.93 | 5.00E-05 | 0.00018571 |
| Zbtb10 | 0.0743503 | 4.24603 | 57.08 | 5.00E-05 | 0.00018571 |
| Bmyc | 0.42139 | 15.0891 | 35.8 | 0.00305 | 0.00760431 |
| Klf4 | 0.412627 | 12.453 | 30.17 | 5.00E-05 | 0.00018571 |
| Six1 | 0.11187 | 3.30063 | 29.5 | 0.0001 | 0.00035325 |
| Hoxb7 | 0.220534 | 5.70191 | 25.856 | 0.00015 | 0.00051198 |
| Sox13 | 0.155761 | 3.73213 | 23.96 | 5.00E-05 | 0.00018571 |
| Sox15 | 0.166005 | 3.97276 | 23.93 | 0.18255 | 0.263107 |
| Ifi205 | 0.438377 | 10.4646 | 23.87 | 5.00E-05 | 0.00018571 |
| Zfp704 | 0.220663 | 4.66521 | 21.14 | 5.00E-05 | 0.00018571 |
| Erg | 3.11126 | 60.7095 | 19.51 | 5.00E-05 | 0.00018571 |
| Cdyl2 | 0.142505 | 2.4963 | 17.517 | 5.00E-05 | 0.00018571 |
| Stat4 | 3.25847 | 54.9235 | 16.85 | 5.00E-05 | 0.00018571 |
| Cebpd | 1.84241 | 25.6114 | 13.8 | 5.00E-05 | 0.00018571 |
| Smad1 | 0.997717 | 12.7398 | 12.77 | 5.00E-05 | 0.00018571 |
| Zfp947 | 0.24179 | 2.65978 | 11 | 5.00E-05 | 0.00018571 |
| Zfp286 | 0.0727163 | 0.796008 | 10.847 | 0.00155 | 0.00414028 |
| Pwwp2b | 0.254013 | 2.77468 | 10.923 | 5.00E-05 | 0.00018571 |
| Sox18 | 0.239009 | 2.49525 | 10.44 | 0.00805 | 0.0177592 |
| Zfp831 | 0.159933 | 1.65848 | 10.37 | 5.00E-05 | 0.00018571 |
| Ccnd1 | 3.49661 | 31.7711 | 9.086 | 5.00E-05 | 0.00018571 |
| Zhx2 | 0.750705 | 6.50422 | 8.664 | 5.00E-05 | 0.00018571 |
| Foxd2 | 0.112663 | 0.919078 | 8.16 | 0.01765 | 0.0351695 |
| Ssbp2 | 1.12133 | 8.19997 | 7.31 | 5.00E-05 | 0.00018571 |
| Hopx | 0.939898 | 6.50938 | 6.9 | 0.00005 | 1.86E-04 |
| Rps6ka5 | 2.03792 | 13.2742 | 6.54 | 5.00E-05 | 0.00018571 |
| Zfp629 | 1.06464 | 6.72112 | 6.312 | 5.00E-05 | 0.00018571 |
| Thrb | 1.27225 | 7.8048 | 6.135 | 5.00E-05 | 0.00018571 |
| Nfam1 | 4.74543 | 28.2802 | 5.955 | 5.00E-05 | 0.00018571 |
| Pyhin1 | 3.57266 | 21.1375 | 5.917 | 5.00E-05 | 0.00018571 |
| Irf8 | 17.0741 | 87.0775 | 5.1 | 5.00E-05 | 0.00018571 |
| Ets1 | 25.7426 | 118.022 | 4.585 | 5.00E-05 | 0.00018571 |
| Zeb2 | 4.15707 | 18.8364 | 4.53 | 5.00E-05 | 0.00018571 |
| Hlx | 1.28521 | 5.74354 | 4.47 | 5.00E-05 | 0.00018571 |
| Hes6 | 20.2404 | 90.0449 | 4.45 | 5.00E-05 | 0.00018571 |
| Kctd1 | 0.715814 | 3.14117 | 4.38 | 5.00E-05 | 0.00018571 |
| Atf3 | 0.858854 | 3.74629 | 4.36 | 5.00E-05 | 0.00018571 |
| Jdp2 | 5.01078 | 21.3202 | 4.257 | 5.00E-05 | 0.00018571 |
| Irf2bp2 | 21.374 | 88.8982 | 4.16 | 5.00E-05 | 0.00018571 |
| Klf3 | 2.25011 | 9.1621 | 4.07 | 5.00E-05 | 0.00018571 |
| Lmo2 | 12.357 | 50.216 | 4.063 | 5.00E-05 | 0.00018571 |
| Elk3 | 6.03004 | 24.4109 | 4.047 | 5.00E-05 | 0.00018571 |
|  |  |  |  |  |  |
| **Stemness** |  |  |  |  |  |
|  |  |  |  |  |  |
| Angptl2 | 0.199299 | 16.3241 | 81.9 | 0.0015 | 0.00402356 |
| Flt3 | 0.779768 | 49.2218 | 63.12 | 5.00E-05 | 0.00018571 |
| Gfra1 | 0.268732 | 14.9358 | 55.58 | 5.00E-05 | 0.00018571 |
| Nlrp1a | 0.0278146 | 1.0297 | 37.01 | 0.00625 | 0.0142738 |
| Kit | 0.779152 | 24.9621 | 32.04 | 5.00E-05 | 0.00018571 |
| Met | 0.211663 | 3.97972 | 18.8 | 5.00E-05 | 0.00018571 |
| Tmem119 | 6.02478 | 88.4283 | 14.672 | 5.00E-05 | 0.00018571 |
| Tifab | 6.43651 | 84.0394 | 13.06 | 5.00E-05 | 0.00018571 |
| Ccl9 | 11.7804 | 139.337 | 11.80 | 0.00005 | 0.00018571 |

**Supplement Table 1:** Summary of gene expression changes in tnFGFR1 expressing cells compared with cells expressing BCR-FGFR1m in the categories of ‘Oncogene’, transcription factor’ and involved in maintaining ‘stemness’. Gene expression (GE) is cited as the mean FPMK values (N-2) in both cases.
